# Long-term effects of zinc oxide nanoparticles on soluble microbial product composition and transformation in submerged membrane bioreactors

**DOI:** 10.1007/s11356-026-38139-3

**Published:** 2026-08-14

**Authors:** Dongqing Zhang, Antoine Prandota Trzcinski

**Affiliations:** 1 Nanyang Environment & Water Research Institute, School of Civil and Environmental Engineering, Nanyang Technological University, 1 Cleantech Loop, #06-10, 637141 Singapore, Singapore; 2 Department of Civil and Environmental Engineering, University of South Florida, 4202E. Fowler Ave, ENB 118, Tampa, FL 33620 USA; 3 School of Science, Engineering and Digital Technologies, University of Southern Queensland, 487 West Street, Toowoomba, 4350 QLD Australia

**Keywords:** Zinc oxide nanoparticles (ZnO NPs), Membrane bioreactor (MBR), Soluble microbial products (SMP), Low-molecular-weight organics, GC–MS

## Abstract

The increasing release of engineered nanoparticles into wastewater systems has raised concerns regarding their long-term effects on biological treatment processes and soluble microbial product (SMP) formation. However, the effects of zinc oxide nanoparticles (ZnO NPs) on the composition, diversity, and persistence of low-molecular-weight (low-MW) SMPs in membrane bioreactors (MBRs) remain poorly understood. In this study, the long-term effects of ZnO NP exposure on SMP characteristics were investigated in submerged anoxic-aerobic MBRs operated with and without ZnO NP addition. SMP production, extracellular polymeric substances (EPS), molecular weight distribution, fluorescence properties, and low-MW SMP identification were analyzed using HPLC-SEC, FEEM fluorescence spectroscopy, and GC–MS. ZnO NP exposure increased polysaccharide production in both loosely bound and tightly bound EPS fractions, indicating stress-induced enhancement of the extracellular protective matrix. High-MW SMP fractions and protein- and fulvic acid-like fluorescence intensities also increased under ZnO NP stress. GC–MS analysis revealed that ZnO NP exposure increased the number and diversity of detectable low-MW SMPs, with a shift toward aromatics, esters, and alcohols, while the relative abundance of alkanes and alkenes decreased. Several compounds were detected exclusively in the ZnO NP-exposed reactor, suggesting stress-induced metabolic transformation and altered biodegradation pathways. Although many SMPs were partially or fully removed during treatment, some recalcitrant compounds persisted in the effluent, including long-chain hydrocarbons and aromatic derivatives. The results demonstrate that ZnO NP exposure substantially alters SMP composition and promotes the formation of more diverse and persistent low-MW organic compounds in MBR systems. These findings provide new insights into nanoparticle-induced stress responses and their implications for SMP dynamics, effluent quality, and membrane fouling in biological wastewater treatment systems.

**Supplementary Information:**

The online version contains supplementary material available at 10.1007/s11356-026-38139-3.

## Introduction

The increasing production and application of engineered nanoparticles (NPs) in industrial, commercial, and consumer products have raised growing concerns regarding their release into aquatic environments and wastewater treatment systems. Among metal oxide nanoparticles, zinc oxide nanoparticles (ZnO NPs) are one of the most widely produced due to their extensive use in cosmetics, coatings, semiconductors, textiles, and antimicrobial products (Franklin et al. 2007; Gottschalk et al. 2009). Wastewater treatment plants (WWTPs) are considered major sinks for ZnO NPs because nanoparticles entering sewer systems are largely retained in activated sludge through adsorption, aggregation, and sedimentation processes (Ma et al. 2013). However, their accumulation in biological treatment systems may affect microbial metabolism and alter the production of soluble microbial products (SMPs), which are closely associated with membrane fouling and effluent quality in membrane bioreactors (MBRs).

Recent studies have emphasized that engineered nanoparticles can influence biological wastewater treatment through both beneficial and adverse pathways. Nanoparticle-based materials have been increasingly explored for adsorption, catalytic oxidation, photocatalysis, membrane modification, and antimicrobial treatment because of their high surface area and tunable reactivity (El Messaoudi et al. 2024). However, when released into activated sludge or MBR systems, metal oxide nanoparticles may also disturb microbial metabolism, sludge aggregation, extracellular polymer formation, and membrane fouling behavior. For example, Ye et al. (2021) reported that ZnO NPs exerted dose-dependent negative effects on sludge flocculation and altered EPS characteristics, while recent reviews have highlighted oxidative stress, metal-ion release, and microbial-community shifts as key mechanisms governing nanoparticle impacts in biological wastewater systems (Zheng et al. 2022; Kedves and Konya 2024). SMPs are soluble organic compounds released during microbial metabolism, substrate utilization, biomass decay, and cell lysis (Sheng et al. 2010). They consist of a heterogeneous mixture of proteins, polysaccharides, humic substances, lipids, organic acids, hydrocarbons, and other metabolites with a wide range of molecular weights and physicochemical properties. In MBR systems, SMPs are widely recognized as major contributors to membrane fouling because they can accumulate on membrane surfaces and within membrane pores, resulting in pore blocking, cake layer formation, and increased transmembrane pressure (Jarusutthirak and Amy 2006). Compared with extracellular polymeric substances (EPS), which are primarily associated with floc surfaces and biofilm matrices, SMPs remain dissolved in the bulk liquid phase and are therefore more mobile and capable of penetrating membrane pores. In particular, low-MW SMPs are considered especially problematic because their small size enables them to pass through or adsorb within membrane pores, contributing to irreversible fouling that is difficult to remove by physical cleaning.

Earlier studies have demonstrated that SMP production and composition are strongly influenced by operational conditions and environmental stressors. Toxic compounds, salinity stress, nutrient limitation, and nanoparticle exposure have all been reported to increase SMP production and alter SMP composition (Wang and Zhang 2010; Zhang et al. 2017; Ye et al. 2021). Under stress conditions, microorganisms often respond by increasing extracellular polymer production and releasing intracellular compounds through oxidative damage and cell lysis. These processes can substantially modify SMP characteristics and influence membrane fouling behavior. Recent studies have also demonstrated that nanoparticle exposure may enhance the accumulation of protein-like and humic-like substances in biological treatment systems (Wang and Chen 2016; Wang et al. 2020). However, most investigations have focused on bulk SMP parameters such as total proteins, polysaccharides, dissolved organic carbon, or fluorescence properties, while the detailed chemical composition of SMPs remains poorly understood.

A multi-analytical approach is required to resolve SMP production and transformation because no single method can capture the full molecular-size range and chemical diversity of dissolved microbial organics. Bulk protein and polysaccharide measurements provide an overall indication of extracellular organic-matter production, whereas high-performance size-exclusion chromatography (HP-SEC) resolves changes in molecular-weight distribution, including the accumulation of high-MW biopolymers that can contribute to membrane fouling. Fluorescence excitation–emission matrix (FEEM) spectroscopy enables rapid characterization of protein-like and humic-/fulvic-like fluorophores, while gas chromatography–mass spectrometry (GC–MS) provides compound-level information for extractable, volatile, and thermally stable low-MW organics. Together, these complementary techniques support a more comprehensive evaluation of dissolved organic matter than bulk parameters alone and can link changes in SMP composition to membrane-fouling propensity and effluent quality. FEEM has been widely used to identify protein-like and humic-like SMP fractions in MBRs (Wang et al. 2009), and HP-SEC has been used to evaluate SMP molecular-weight distributions and the accumulation of high-MW foulants (Wang and Zhang 2010). At the molecular level, GC–MS and LC–MS have enabled identification of specific SMP compounds and transformation products. For example, Trzcinski and Stuckey (2010) identified recalcitrant low-MW compounds in anaerobic MBR effluents, while Kunacheva et al. (2017) demonstrated that hydraulic retention time influenced the composition and biodegradability of low-MW SMPs in submerged anaerobic MBRs. Zhang et al. (2017) further reported that environmental stress promoted the formation of diverse low-MW compounds associated with biomass decay and metabolic transformation.

Despite progress in nanoparticle-enabled remediation and in SMP characterization, the environmental consequences of nanoparticle release into biological wastewater-treatment systems remain insufficiently resolved. Most studies of ZnO NPs in activated sludge and MBRs have focused on bulk treatment performance, nitrogen removal, microbial-community shifts, oxidative stress, or total EPS/SMP concentrations. Consequently, it remains unclear whether long-term ZnO NP exposure changes the molecular composition, diversity, and persistence of low-MW SMPs that are potentially mobile in the treated effluent. This limitation is important because conventional bulk indicators cannot distinguish whether elevated SMP concentrations arise from active extracellular secretion, altered metabolic pathways, biomass turnover, or the release of intracellular constituents following cell damage.

Low-MW SMPs warrant particular attention because they are soluble and may remain in the liquid phase during membrane filtration, thereby influencing permeate quality and downstream receiving-water environments. Their chemical composition can also provide molecular-level indicators of altered lipid turnover, protein degradation, and secondary metabolism under nanoparticle stress. Although ZnO NPs can induce oxidative stress through particle–cell interactions and Zn^2+^ release, evidence linking long-term ZnO NP exposure to specific low-MW SMP transformations across sequential anoxic, aerobic, and membrane-separated treatment stages is scarce. Addressing this gap is necessary to evaluate the environmental compatibility of nanoparticle-containing waste streams and to support the design of robust, sustainable MBR systems.

Accordingly, this study investigated the long-term effects of stepwise ZnO NP exposure on SMP formation and transformation in parallel submerged anoxic-aerobic MBRs. The novelty of this work lies in combining a long-term parallel-MBR exposure design with a complementary and practically accessible analytical workflow comprising EPS quantification, HP-SEC, FEEM fluorescence spectroscopy, and GC–MS. This approach provides multi-scale evidence of ZnO NP-associated SMP transformation, from bulk extracellular polymers and molecular-weight distribution to fluorescent fractions and extractable low-MW compounds. The use of an untreated control MBR operated in parallel with the ZnO NP-exposed MBR over 144 days further strengthens the reliability of the comparison. Importantly, the approach links molecular changes in SMPs to performance-relevant outcomes, including the potential formation of more persistent dissolved organics that may compromise effluent organic-matter quality and contribute to membrane fouling. Unlike studies limited to total SMP/EPS concentrations or reactor-performance indicators, the present work evaluates whether nanoparticle stress is associated with changes in molecular-weight distribution, fluorescent organic-matter fractions, chemical diversity, and the apparent persistence of individual low-MW compounds.

Specifically, the study aimed to (i) quantify the effects of ZnO NPs on SMP and EPS production, (ii) determine changes in SMP molecular-weight distribution and fluorescence characteristics, (iii) identify and compare extractable low-MW SMP compounds in control and ZnO NP-exposed MBRs, and (iv) assess the apparent persistence of identified compounds across the treatment train. The results provide a molecularly resolved basis for evaluating nanoparticle-related risks to MBR effluent quality and membrane operation. They may also inform future monitoring strategies and the design of pretreatment or source-control measures for nanoparticle-containing wastewater streams, supporting safer implementation of nanotechnology within sustainable water-management systems. The results may also support future development of molecular indicators for nanoparticle stress and operational strategies to protect effluent quality and membrane performance in MBRs treating nanoparticle-containing wastewater.

## Materials and methods

### Setup and operation of MBR systems

Two identical lab-scale MBR systems, i.e., MBR-C (control) and MBR-T (treatment), consisting of an anoxic reactor (3 L) followed by an aerobic reactor (7 L) were operated continuously at ambient temperature (24–26 °C) (Fig. S1). A hollow fiber ultrafiltration membrane (ZeeWeed 500, GE Singapore) made of polyvinylidene fluoride with an effective membrane surface area of 565 cm^2^ and a nominal pore size of 0.04 μm was submerged in the aerobic tank. The inoculum for both reactors was mixed liquor suspended solids (MLSS) obtained from Ulu Pandan Wastewater Reclamation Plant (WRP), Singapore. The concentrations of the feed components in synthetic wastewater were as described by Zhang et al. (2017) (in mg L^−1^): 356 sucrose (C_12_H_22_O_11_), 22 KH_2_PO_4_, 153 NH_4_Cl, 34 CaCl_2_, 640 NaHCO_3_, 30 CuSO_4_·5H_2_O, 20 MgSO_4_·7H_2_O, 20 FeSO_4_·7H_2_O, 58 CoCl_2_·6H_2_O, 150 H_3_BO_4_, 20 EDTA (C_10_H_16_N_2_O_8_), and 30 KI. The synthetic wastewater used in this study was designed to simulate typical municipal wastewater, containing a mixture of carbon (e.g., sucrose), nitrogen (e.g., NH_4_^+^–N), phosphorus (e.g., PO_4_^3^⁻–P), and essential trace elements. The use of synthetic wastewater allows for controlled and reproducible conditions, minimizing variability associated with real wastewater and enabling clearer interpretation of the effects of ZnO nanoparticles on microbial processes and SMP production. The MLSS concentration in both reactors was maintained at approximately 3–6 g L^−1^ through controlled sludge wasting (SRT = 25 d), and no significant difference was observed between the control and ZnO NP-dosed reactors. The hydraulic retention time (HRT) of 15 h was selected to provide stable biological treatment and sufficient contact time for carbon and nutrient removal. Although full-scale municipal MBRs are commonly operated at shorter HRTs (typically 4–10 h), laboratory-scale studies frequently employ HRTs of 10–24 h to maintain stable microbial communities, minimize operational variability, and facilitate mechanistic investigations of stress responses, including the effects of emerging contaminants and nanoparticles (Meng et al. 2009; Rahman et al. 2023). Therefore, the selected HRT represents a controlled laboratory operating condition intended to investigate microbial adaptation rather than directly replicate full-scale operation.

Both reactors were operated for three months to achieve steady state. Thereafter, the ZnO NPs were added into the influent wastewater to achieve a final concentration of 1.0 mg L^−1^ from day 1, 10 mg L^−1^ from day 49 and 50 mg L^−1^ from day 96. The operation of two MBRs for the experiment lasted 144 days. The control reactor (MBR-C) only received regular feed without any ZnO NPs during the entire experiment. The stepwise increase in ZnO NPs concentration from 1 to 10 mg L^−1^ and then to 50 mg L^−1^ was designed to simulate progressive exposure and allow microbial acclimation prior to higher stress conditions. However, this approach does not fully decouple time-dependent adaptation from dose-dependent effects. Therefore, the observed responses should be interpreted as the combined outcome of prolonged exposure and increased nanoparticle concentration.

### Characterization of ZnO NPs

The ZnO NPs (50% w/w) were purchased from Sigma-Aldrich (Singapore), and stock solutions (100 mg L^−1^) were prepared. To minimize ZnO NP aggregation, ultrasonic treatment (30 °C, 100 W, 40 kHz) for 30 min and shaking for 2 h was carried out to enhance their dispersion before the ZnO NP suspension was used. The morphology and primary particle-size distribution of the ZnO NPs were examined by transmission electron microscopy (TEM-JEOL JEM-3010, Japan) (Fig. S2). The particles ranged from 15 to 47 nm, with a mean diameter of 33 ± 8 nm (*n* = 107). Particle size analysis was performed using a Zetasizer Nano Z90 (Malvern Instruments, UK). Although only primary particle size and morphology were characterized in the present study, previous work using the same ZnO NPs and reactor system (Zhang et al. 2017) demonstrated that ZnO NPs undergo partial dissolution in mixed liquor, resulting in measurable Zn^2+^ concentrations and that aggregation occurs due to interactions with biomass and EPS. These processes influence nanoparticle bioavailability and contribute to the observed biological effects.

### Determination of protein and polysaccharide concentrations in EPS

The extraction and characterization of SMPs followed the procedure described by Zhang et al. (2017). Mixed liquor samples were collected from the reactor and centrifuged at 6000 g for 10 min. The resulting supernatant was used for SMP analyses. The dewatered sludge pellet was washed with 0.9% NaCl solution (w/w) and then sonicated at 20 kHz for 2 min and centrifuged at 8,000 g for 10 min. The supernatant was considered loosely bound EPS (LB-EPS); the residual sludge pellet left in the centrifuge tube was suspended in NaCl solution, heated at 60 °C for 1 h, and centrifuged at 11,000 g for 10 min. The supernatant was regarded as tightly bound EPS (TB-EPS).

### SMP molecular weight (MW) distribution

A high-performance size-exclusion chromatograph (HP-SEC) (1260 LC system, Agilent, Santa Clara, CA, USA) equipped with a PL Aquagel-OH 8 lm MIXED-M column was used to determine the overall MW distribution of organic compounds. Milli-Q water was used as the mobile phase at a flow rate of 1 mL min^−1^. Polyethylene glycols (PEGs) and polyethylene oxide standards with molecular weights of 500 kDa, 70 kDa, 4 kDa, 600 Da, and 106 Da were used to calibrate the column. MW was calculated based on a linear relationship between the log of MW (Da) and retention time (Rt in min) as shown in Eq. (1):

1 $$\log\;\left(MW\right)=9.883-0.6748\,\left(Rt\right)$$

### Three-dimensional fluorescence excitation emission matrix (FEEM)

Three-dimensional fluorescence excitation-emission matrix (FEEM) spectra were measured using a luminescence spectrometer (LS55, Perkin-Elmer®, Waltham, MA, USA). The spectrometer slits were set at 10 nm for both excitation and emission, and excitation wavelengths were increased from 220 to 600 nm in 10 nm steps; for each excitation wavelength, the emission was detected from 300 to 550 nm in 10 nm steps. The spectra were recorded at a scan rate of 10,000 nm min^−1^, using excitation and emission slit bandwidths of 10 nm, and the software FL Winlab Version 4.00.03 (Perkin Elmer) was used to handle the EEM data.

### Identification and characterization of low-MW SMPs

Low-MW SMPs were specifically targeted because they are more soluble, more bioavailable, and more likely to pass through membrane systems into the effluent, thereby having a greater impact on effluent quality and potential downstream environmental effects. Low-MW compounds can be reliably identified using GC–MS, allowing for detailed compositional analysis. In contrast, high-MW SMPs are typically retained by the membrane and are more commonly characterized using bulk parameters or advanced techniques. In order to further investigate the impacts of ZnO NPs on SMPs, GC–MS was used to identify and characterize non-polar, volatile, and thermally stable low-MW (< 580 Da) SMPs in the MBRs, with and without the addition of 50 mg L^−1^ ZnO NPs. Samples for GC–MS analysis were collected from the anoxic tank, aerobic tank, and effluent at steady-state conditions. All analyses were conducted in triplicate. Liquid–liquid extraction was carried out on 100 mL of filtered supernatant (< 0.45 μm) using 70 mL dichloromethane (GC–MS grade, Merck), following (Wu and Zhou 2010). The two phases were mixed for 3 min, and then separated over 5 min; traces of water were removed by mixing the solvent phase with Na_2_SO_4_. The solvent was evaporated at 50 °C under vacuum until 1 mL remained, and the samples were analyzed using a gas chromatograph (5890 Series) equipped with a QP2010Ultra Mass Spectrometry Detector (GCMS-QP2010ULTRA, Shimadzu, Japan). The analytes were separated using an RTX®−5MS column (30 m × 0.25 mm ID, Restek) for low-to-mid polarity compounds. Splitless injection was used with a column temperature of 280 °C, while helium was used as the carrier gas at a column flow rate of 1 mL min^−1^. The MS was operated in the electron impact ionization mode (70 eV). The total run time per sample was 60 min, and the GC–MS oven temperature was 50 °C, hold 7 min, increase at 7 °C min^−1^ to 325 °C, and then hold for 14 min. The temperature program was modified from this for the alkane standards (C_10_–C_40_, 50 mg L^−1^, Sigma-Aldrich); the MS was operated in the electron ionization mode (EI) with the ion source temperature at 230 °C, and mass spectra were acquired from *m/z* 30 to 580 after a 10 min solvent cut time. Chromatographic peaks were analyzed using the NIST11 library (National Institute of Standards and Technology, Gaithersburg, MD, USA, http://www.nist.gov/srd/mslist.htm), and the compound was considered identified if the match percentage was higher than 80%; compounds below 80% were described as unknown peaks. The similarity index, mass spectrum, and retention index were used to evaluate candidate compounds suggested by the NIST library. Method blanks (deionized water) were run through the same pretreatment and analysis, while feed samples were also run to identify compounds in the feed. Alkanes were selected for the approximate quantification of SMPs since alkanes have widely varying chain lengths (C_10_–C_40_) and hence can cover most of the volatility range of the RTX®−5MS column (Supplementary Table S1). In this study, compounds detected in the effluent were operationally defined as recalcitrant, while those showing partial reduction in relative peak area across treatment stages were considered slowly biodegradable. This classification is qualitative and based on persistence through the treatment process, consistent with previous studies on SMP characterization (e.g., Trzcinski and Stuckey 2010; Kunacheva et al. 2017). The selected analytical methods were intended to provide complementary information on SMP quantity, molecular-size distribution, fluorescence-active fractions, and extractable low-MW compounds. FTIR (Fourier-Transform Infrared Spectroscopy), FESEM (Field-Emission Scanning Electron Microscopy), and ^1^H NMR (proton nuclear magnetic resonance) were not included because the study did not aim to characterize nanoparticle surface chemistry, particle deposition on sludge or membrane surfaces, or the complete structural composition of purified SMP fractions. These complementary techniques could be applied in future work to further resolve functional-group changes and nanoparticle–biomass interactions.

### Statistical analysis

Quantitative EPS and SMP measurements were performed in triplicate and are presented as mean ± standard deviation (SD). Differences among the control, 1 mg L⁻^1^ ZnO NP, and 50 mg L⁻^1^ ZnO NP conditions were evaluated using one-way analysis of variance (ANOVA). Statistical significance was accepted at *p* < 0.05. Statistical analyses were performed using SPSS Statistics 20.

## Results and discussion

### SMP production

**Fig. 1:**
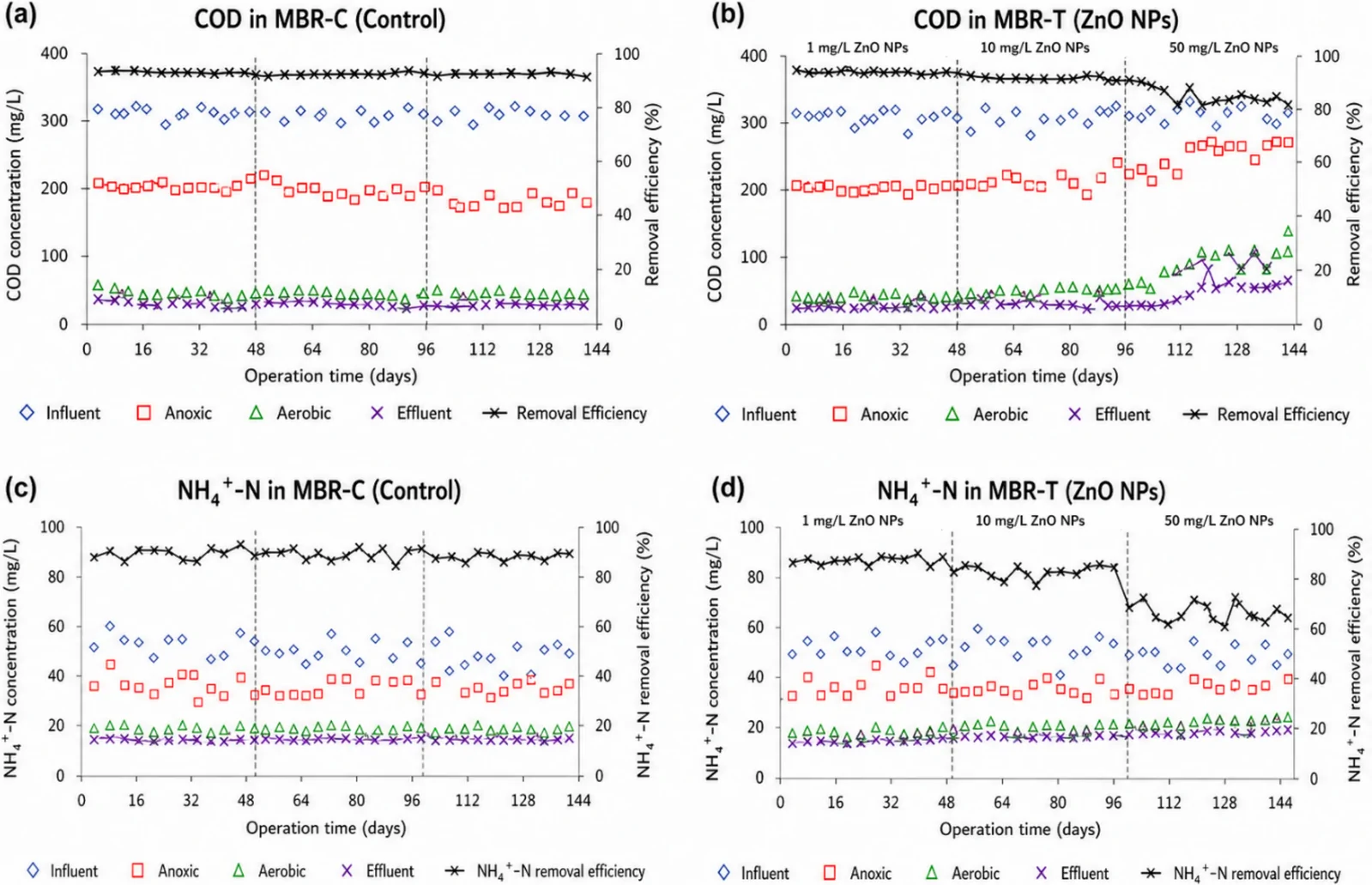
Pollutant-removal performance of the MBRs: **a** COD removal in MBR-C; **b** COD removal in MBR-T; **c** NH_4_^+^–N removal in MBR-C; and **d** NH_4_^+^–N removal in MBR-T. MBR-C denotes the control reactor, and MBR-T denotes the ZnO NP-exposed reactor. Values are presented as means of triplicate measurements

The effects of ZnO NPs on MBR performance are shown in Fig. 1. Although exposure to 1 mg L^−1^ ZnO NPs did not result in significant (*p* > 0.05) changes in removal efficiencies, 10 mg L^−1^ ZnO NPs decreased chemical oxygen demand (COD) removal substantially from 93.1 to 90.1%. In the presence of 1 mg L^−1^ ZnO NPs, the relatively stable NH_4_^+^-N removal of 85.0 ± 2.5% indicated that this ZnO NP concentration was within the tolerance range of the activated sludge. However, with 10 and 50 mg L^−1^ ZnO NPs, removal decreased significantly to 78.2 ± 5.8% (*p* < 0.05) and 65.2 ± 5.7% (*p* < 0.05), respectively (Fig. 1). No significant difference in MLSS concentration was observed between the control and ZnO NP-dosed reactors during steady-state operation.

In addition to the changes observed in LB-EPS, the tightly bound EPS (TB-EPS) fraction also exhibited notable variations under ZnO NP exposure (Table 1), which provide further insight into the protective mechanisms of microbial aggregates. Compared with the control reactor, LB-EPS proteins increased from 27.8 ± 2.4 to 36.3 ± 4.7 mg g^−1^ VSS (Volatile Suspended Solids) (*p* < 0.05), while polysaccharides increased from 7.6 ± 1.1 to 12.1 ± 1.8 mg g^−1^ VSS at 50 mg L^−1^ ZnO NP exposure (*p* < 0.05). Compared to LB-EPS, TB-EPS is more closely associated with cell surfaces and contributes to the structural integrity and stability of flocs and biofilms. Under ZnO NP stress, both protein and polysaccharide contents increased in the TB-EPS fraction, indicating enhanced production of extracellular protective materials in response to nanoparticle-induced stress (Table 1). Compared with the control reactor, TB-EPS proteins increased from 76.1 ± 5.3 to 105.3 ± 8.5 mg g^−1^ VSS (*p* < 0.05), while polysaccharides increased from 42.7 ± 3.8 to 54.3 ± 4.6 mg g^−1^ VSS at 50 mg L^−1^ ZnO NP exposure (*p* < 0.05). The stronger increase observed for proteins suggests that proteinaceous components played a major role in the microbial stress response, potentially through metal binding, enzymatic activity, and stabilization of the extracellular matrix. At the same time, the elevated polysaccharide content likely contributed to the formation of a denser diffusion-limiting barrier capable of adsorbing nanoparticles and complexing dissolved Zn^2+^ ions. Together, these changes indicate that microorganisms reinforced the TB-EPS matrix as a protective mechanism to reduce direct cellular exposure to ZnO NPs and associated oxidative stress.

**Table 1:** Effects of long-term ZnO NP exposure on the production of soluble microbial products (SMPs) and loosely bound and tightly bound extracellular polymeric substances (EPS) in submerged MBR systems

	LB-EPS (mg g^−1^ VSS)		TB-EPS (mg g^−1^ VSS)		SMP (mg L^−1^)
	Protein	Polysaccharide	Protein	Polysaccharide	
Control	27.8 ± 2.4	7.6 ± 1.1	76.1 ± 5.3	42.7 ± 3.8	15.9 ± 1.2
1 mg L^−1^ ZnO NPs	29.4 ± 3.9	8.8 ± 1.3	83.8 ± 6.8	45.5 ± 3.1	28.8 ± 3.1*
50 mg L^−1^ ZnO NPs	36.3 ± 4.7*	12.1 ± 1.8 *	105.3 ± 8.5*	54.3 ± 4.6*	43.2 ± 3.7*

* means significantly different from the control (*p* < 0.05)

The observed changes in TB-EPS composition are consistent with a stress-induced reinforcement of the extracellular matrix, where increased polysaccharide production enhances floc cohesion and provides a more effective physical and chemical barrier against nanoparticle penetration. This protective effect is particularly important in biofilm systems, where TB-EPS constitutes the dominant matrix component. In contrast to the more dynamic and loosely associated LB-EPS fraction, TB-EPS reflects longer-term structural adaptation of the microbial community. Therefore, the combined analysis of LB-EPS and TB-EPS suggests that ZnO NP exposure triggers both immediate (LB-EPS) and sustained (TB-EPS) protective responses, contributing to microbial resilience under nanoparticle stress. SMP generation was inferred indirectly from increases in SMP concentration (measured as total SMP, protein, and polysaccharides) and changes in compound diversity under ZnO NP exposure. This approach did not distinguish between active secretion and release due to cell lysis.

These findings are consistent with recent studies showing that nanoparticle exposure can modify extracellular polymer production and sludge physicochemical properties. Ye et al. (2021) observed that ZnO NP exposure altered EPS composition and reduced sludge flocculation performance, indicating disruption of microbial aggregate stability. Similarly, recent reviews have noted that nanoparticle-induced oxidative stress, particle–cell interactions, and metal-ion release can stimulate microbial defensive responses, including EPS secretion and changes in soluble organic matter production (Zheng et al. 2022; Kedves and Konya 2024). The increase in EPS and SMP concentrations observed in the present study therefore supports the interpretation that long-term ZnO NP exposure triggered a protective extracellular response and enhanced biomass turnover in the MBR system.

**Fig. 2:**
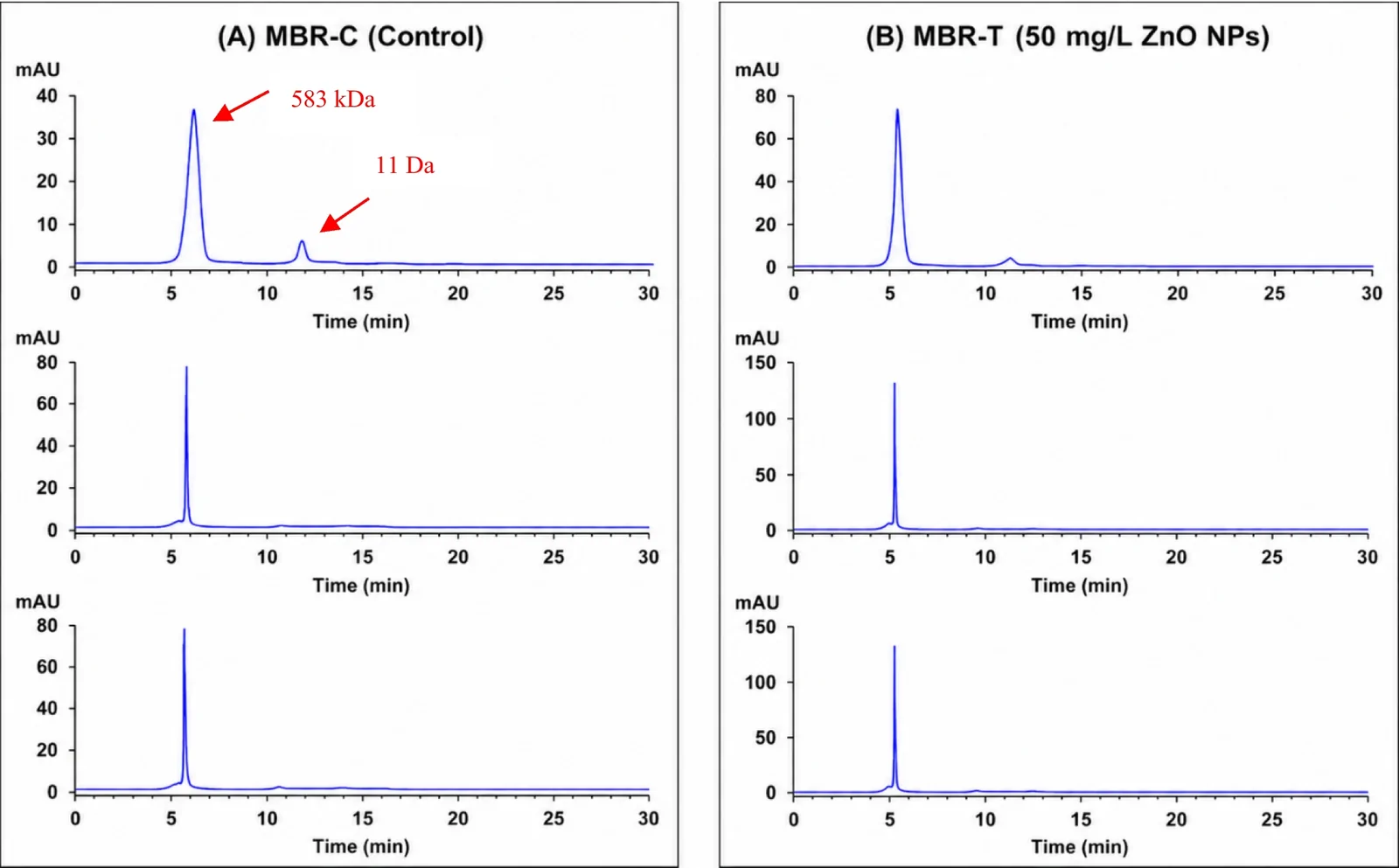
The molecular weight (MW) distribution of SMPs using high-performance size-exclusion chromatograph (HP-SEC): **A** MBR-C without the addition of ZnO NPs and **B** MBR-T under exposure to 50 mg L^−1^ ZnO NPs. “C” represents control; and “T” represents treatment. Top: anoxic tank, middle: aerobic tank, bottom: effluent

HPLC-SEC analysis revealed pronounced changes in the molecular weight distribution of SMPs following long-term ZnO NP exposure (Fig. 2). In the control reactor (MBR-C), the SMP profile was dominated by relatively low- to intermediate-MW fractions, whereas exposure to 50 mg L^−1^ ZnO NPs resulted in a marked increase in high-MW compounds, particularly around 583 kDa in the anoxic compartment, where the signal intensity increased from approximately 35 to 70 mAU. Similar trends were also observed in the aerobic compartment and, to a lesser extent, in the effluent, indicating that ZnO NP exposure promoted the accumulation of larger soluble biopolymeric substances throughout the treatment process.

The increase in high-MW SMP fractions is commonly associated with stress-induced secretion of extracellular materials and the release of intracellular macromolecules following membrane damage and partial cell lysis (Aquino and Stuckey 2004; Wang and Zhang 2010). Under nanoparticle stress, microorganisms often produce additional extracellular proteins and polysaccharides as protective barriers against oxidative stress and metal toxicity. This interpretation is consistent with the elevated EPS concentrations observed in Table 1, particularly the marked increase in TB-EPS proteins and polysaccharides under ZnO NP exposure. High-MW SMPs are generally composed of protein-like substances, polysaccharides, humic-like compounds, and soluble biopolymers derived from microbial metabolism and biomass decay (Sheng et al. 2010).

The pronounced accumulation of high-MW compounds in the anoxic compartment may indicate that anaerobic and facultative microorganisms were more sensitive to ZnO NP stress or less capable of degrading released macromolecules compared with the aerobic biomass. Reduced biodegradation efficiency under stress conditions may have contributed to the persistence of these large soluble compounds. Previous studies have shown that high-MW SMP fractions are less biodegradable and more likely to accumulate in MBR systems under unfavorable operating conditions (Ni et al. 2009; Kunacheva et al. 2017).

**Fig. 3:**
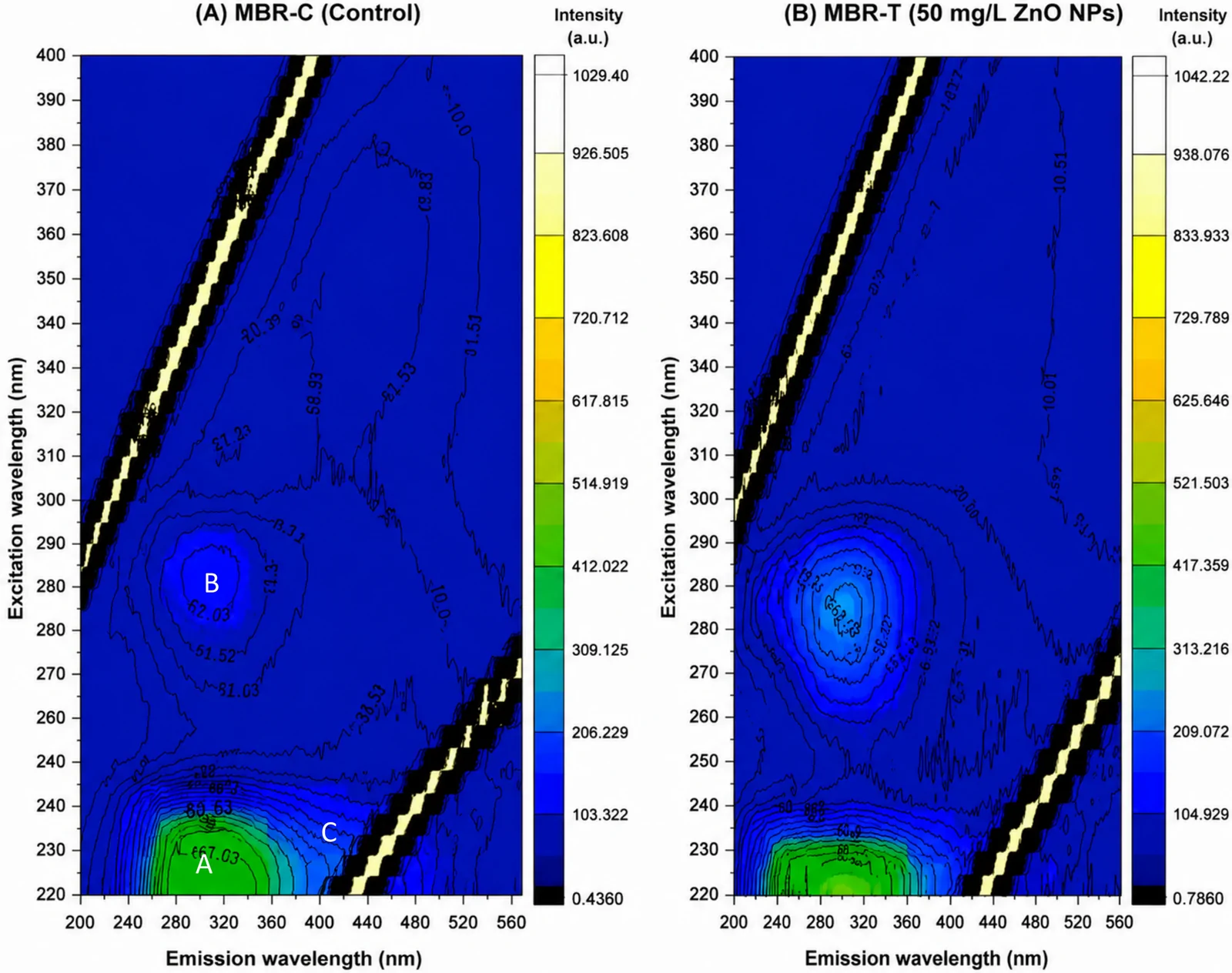
Three-dimensional fluorescence excitation-emission matrix (FEEM) contour maps of SMPs in MBR-C and MBR-T. **A** aerobic MBR-C and **B** aerobic MBR-T at 50 mg L^−1^ ZnO NPs concentration. “C” represents control; and “T” represents treatment

FEEM analysis provided further insight into the compositional changes of SMPs under ZnO NP exposure (Fig. 3). Three major fluorescence regions were identified in both reactors. Region A (Ex/Em = 225/360 nm) corresponded mainly to aromatic protein-like substances, region B (Ex/Em = 275/350 nm) was associated with tryptophan and tryptophan-like protein compounds, and region C (Ex/Em = 240/400 nm) represented fulvic acid-like substances (Wang et al. 2009). Compared with the control reactor, the fluorescence intensities of all three regions increased markedly in MBR-T exposed to 50 mg L^−1^ ZnO NPs, indicating enhanced production and accumulation of soluble organic matter under nanoparticle stress.

The increased fluorescence intensities observed in regions A and B indicate enhanced accumulation of proteinaceous SMPs, which are commonly associated with microbial stress responses, biomass decay, and release of intracellular materials following membrane damage (Sheng et al. 2010). Protein-like SMPs are known to increase under unfavorable environmental conditions because microorganisms often excrete extracellular proteins and enzymes as protective or adaptive responses against toxic compounds (Wang and Zhang 2010). Oxidative stress induced by ZnO NPs and released Zn^2+^ ions may have promoted partial cell lysis, thereby releasing intracellular proteins and amino acid-derived compounds into the mixed liquor. This interpretation is consistent with the elevated protein concentrations observed in both LB-EPS and TB-EPS fractions (Table 1).

The enhanced fluorescence intensity observed in region C indicates increased production or accumulation of fulvic acid-like substances in the ZnO NP-exposed reactor. Fulvic-like compounds are generally considered more humified and recalcitrant than protein-like SMPs and are often associated with microbial degradation products and long-term organic matter transformation processes (Baker 2001). Their accumulation under ZnO NP exposure may therefore indicate incomplete biodegradation and enhanced formation of refractory dissolved organic matter. Similar increases in humic- and fulvic-like fluorescence signals have previously been reported under toxic or oxidative stress conditions in biological wastewater treatment systems (Wang et al. 2020).

The EPS, FEEM, and HPLC-SEC results indicate that ZnO NP exposure promoted the accumulation of larger and more complex soluble organic compounds through combined effects of microbial stress responses, enhanced extracellular polymer secretion, and biomass decay. These compositional changes are particularly important for MBR operation because high-MW proteins, polysaccharides, and humic-like substances are known to contribute to membrane fouling through cake layer formation, pore blocking, and increased filtration resistance (Jarusutthirak and Amy 2006). Therefore, prolonged ZnO NP exposure may not only alter microbial metabolism but also significantly influence SMP fouling characteristics and effluent organic matter composition in MBR systems.

### Low-MW (< 580 Da) SMPs in the MBR without the addition of ZnO NPs

**Fig. 4:**
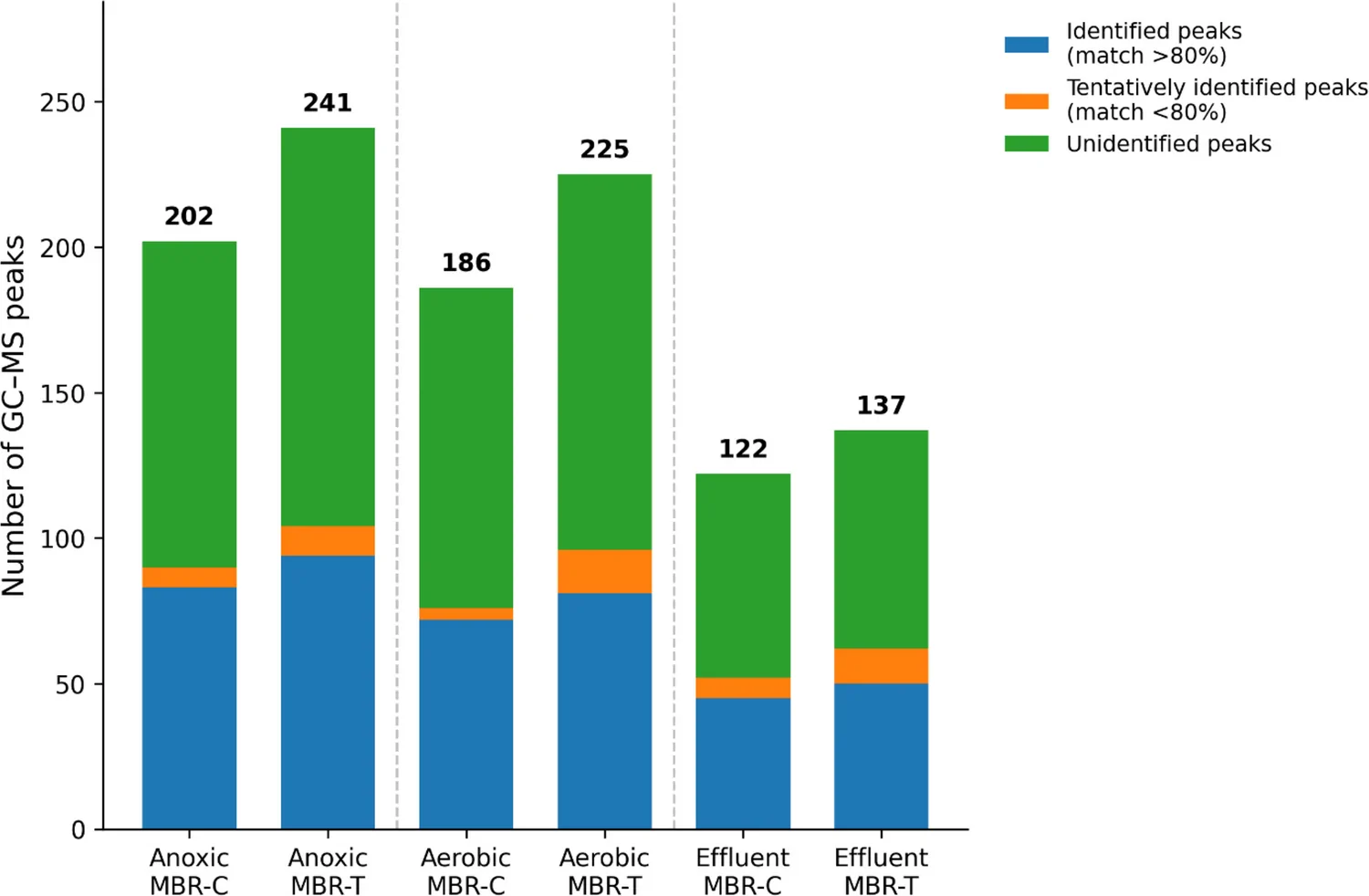
GC–MS peak distribution of low-MW SMPs in MBR-C and MBR-T. Stacked bars show the number of identified peaks with match quality > 80%, tentatively identified peaks with match quality < 80%, and unidentified peaks in the anoxic, aerobic, and effluent samples. Numbers above bars indicate total detected peaks. MBR-C: control reactor; MBR-T: ZnO NP-dosed reactor

GC–MS analysis revealed the presence of a diverse range of low-molecular-weight (low-MW, < 580 Da) SMPs in the control membrane bioreactor (MBR-C) (Fig. 4). A greater number of total GC–MS peaks was detected in the anoxic compartment (202 peaks) compared with the aerobic compartment (186 peaks), suggesting that the anoxic stage generated a more chemically diverse pool of soluble metabolites. This difference may be associated with the distinct metabolic pathways occurring under anoxic conditions, where fermentation and incomplete substrate degradation can produce a broader variety of intermediate organic compounds.

Among the detected peaks, a large proportion corresponded to unidentified compounds, indicating the complexity of SMP composition and the limitations of existing spectral databases for characterizing wastewater-derived organic matter. Nevertheless, a substantial number of compounds were successfully identified with spectral match qualities greater than 80%, demonstrating that the low-MW SMP fraction consisted of a mixture of hydrocarbons, alcohols, esters, aromatic compounds, ketones, and other soluble metabolites.

The number of detectable low-MW compounds was lower in the effluent compared with both biological compartments, indicating that a portion of the SMPs generated during biological treatment was either biodegraded, retained within the system, or removed by membrane filtration. However, several compounds remained detectable in the effluent, demonstrating that certain low-MW SMPs were relatively persistent during treatment. These residual compounds may contribute to effluent dissolved organic matter and potentially influence downstream water quality and membrane fouling behavior.

**Fig. 5:**
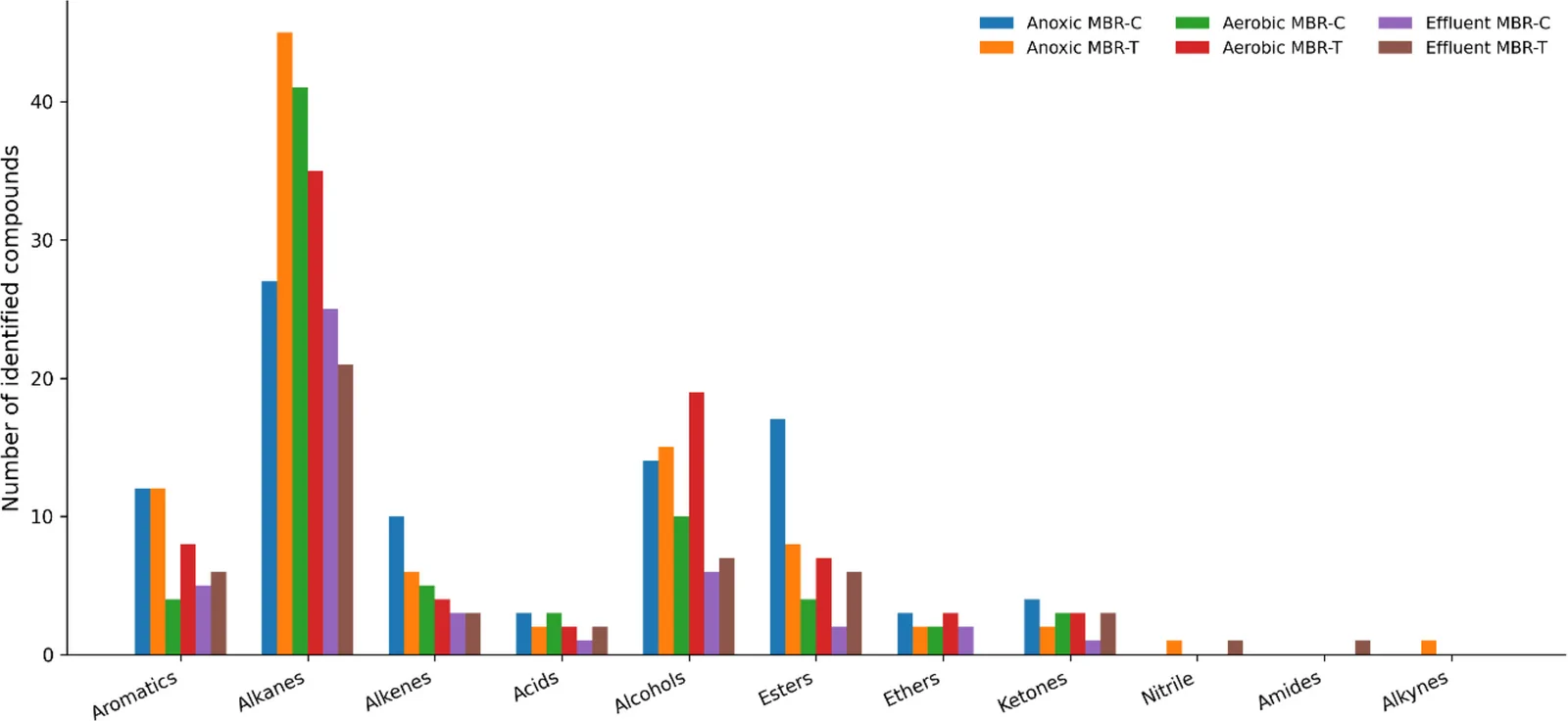
Distribution of low-MW SMP compound classes identified by GC–MS in MBR-C and MBR-T. Comparison of compound classes detected in the anoxic, aerobic, and effluent samples from the control reactor (MBR-C) and ZnO NP-dosed reactor (MBR-T)

In the aerobic compartment, 72 compounds were identified, representing approximately 39% of the total detected peaks. The identified compounds were dominated by alkanes, which accounted for 57% of the total identified compounds, followed by alcohols (14%), alkenes (7%), aromatics (6%), and esters (6%) (Fig. 5). Smaller proportions of ketones, ethers, acids, and other compound classes were also detected.

The predominance of alkanes suggests that hydrocarbon-like compounds represented a major fraction of the low-MW SMP pool under normal operating conditions. These compounds are commonly associated with lipid degradation, microbial metabolism, and the breakdown of cellular materials during biological treatment. Alcohols and esters may originate from fatty acid metabolism and intermediate biodegradation pathways, whereas aromatic compounds likely reflect the partial transformation of more complex organic matter.

Compared with the aerobic compartment, fewer compounds were identified in the effluent, with only 45 compounds showing match qualities greater than 80% (Fig. 4). This decrease indicates that a substantial portion of the low-MW SMPs generated during biological treatment was either biodegraded, transformed, or retained within the membrane bioreactor system before discharge. Nevertheless, several compounds persisted in the effluent, demonstrating that certain low-MW SMP fractions were relatively resistant to biological degradation and membrane retention.

The distribution of compound classes in the effluent remained broadly similar to that observed in the aerobic compartment (Fig. 5). Alkanes continued to dominate the identified compounds (56%), followed by alcohols (13%), aromatics (11%), and alkenes (7%). However, the relative increase in aromatic compounds in the effluent suggests that aromatic SMPs were more recalcitrant than many aliphatic compounds during treatment. Aromatic structures are generally more resistant to microbial degradation due to their stable ring structures and lower biodegradability compared with linear hydrocarbons.

An additional observation was the detection of nitrile, amide, and alkyne compounds exclusively in the ZnO NP-exposed reactor (Fig. 5). Their absence in the control reactor should not be interpreted as complete absence from the biological system, but rather that their concentrations were below the GC–MS detection limit under normal operating conditions. Their occurrence following ZnO NP exposure suggests that nanoparticle-induced oxidative stress promoted alternative metabolic pathways and transformation reactions associated with biomass turnover and degradation of intracellular constituents. Nitriles and amides can be formed as intermediates during the degradation of nitrogen-containing biomolecules, whereas unsaturated hydrocarbons such as alkynes may arise from secondary oxidation and fragmentation of lipids or other organic compounds. Similar stress-induced changes in dissolved organic matter composition have been reported following exposure of activated sludge microorganisms to engineered nanoparticles and other environmental stressors, where oxidative damage and altered microbial metabolism generated new low-molecular-weight metabolites rather than entirely new biochemical pathways (Sheng et al. 2010; Wang et al. 2020; Gao et al. 2025).

Overall, Figs. 4 and 5 demonstrate that the control MBR produced a chemically diverse mixture of low-MW SMPs, with compound composition evolving across treatment stages. Although many compounds were partially removed during treatment, several persistent low-MW SMPs remained detectable in the effluent, highlighting the complexity and incomplete biodegradation of soluble organic matter in MBR systems even under non-stressed conditions.

Supplementary Tables S2, S3, S4. summarize the low-MW SMPs identified in the anoxic, aerobic, and effluent samples of the control reactor (MBR-C). One of the key findings from these tables was the detailed identification of compounds that were fully biodegradable, slowly biodegradable, or recalcitrant during the MBR treatment process. This molecular-level characterization provides important insight into the fate of low-MW SMPs during biological treatment and membrane filtration.

Several compounds detected in the anoxic and aerobic compartments were absent from the effluent, indicating effective removal during treatment. These compounds included alcohols such as 1-dodecanol, 2-octyl-, 1-tetradecanol, n-nonadecanol-1, and octacosanol; acids such as n-hexadecanoic acid; esters such as isopropyl palmitate; and ethers such as tetraethylene glycol monododecyl ether. Their disappearance from the effluent suggests that these compounds were either readily biodegradable, retained within the biomass or cake layer, or effectively rejected by the membrane system. In parallel, the number of aromatic compounds decreased from 12 in the anoxic stage to only 5 in the permeate (Fig. 5), indicating partial biodegradation of aromatic hydrocarbons during treatment. Moreover, the maximum molecular weight of compounds detected decreased from 506 Da in the aerobic stage to 394 Da in the permeate, suggesting preferential removal of larger low-MW compounds during biological treatment and membrane filtration.

Despite this removal, several compounds persisted throughout the treatment process and were still detected in the permeate. In this study, compounds were considered recalcitrant when they remained detectable in all three compartments (anoxic, aerobic, and permeate), indicating persistence through both biological treatment stages and membrane separation. A total of 22 compounds met this criterion (Table 2). These compounds were primarily composed of long-chain alkanes, branched hydrocarbons, alcohols, aromatic compounds, and phthalate derivatives.

**Table 2:** Low-MW SMP compounds detected throughout the MBR-C treatment process and classified as slowly biodegradable or recalcitrant based on their persistence and removal behavior

Compound class	Compound name	RI	MW (Da)	Formula	Similarity (%)	Relative area (%)	Removal (%)
Alkanes/hydrocarbons	Dodecane, 4,6-dimethyl-	1285	198	C_14_H_30_	94	0.95	6.1
	Nonane, 5-butyl-	1249	184	C_13_H_28_	91	0.17	81.9
	Tridecane	1304	182	C_13_H_26_	95	0.65	< 0
	Pentadecane	1512	212	C_15_H_32_	90	1.34	5.1
	Tetradecane	1413	198	C_14_H_30_	95	0.52	< 0
	Heptadecane	1711	240	C_17_H_36_	91	1.41	< 0
	10-Methylnonadecane	1945	282	C_20_H_42_	83	2.25	41.9
	1-Nonadecene	1900	266	C_19_H_38_	93	0.69	37.6
	Nonadecane	1910	268	C_19_H_40_	90	1.62	57.5
	Heptadecane, 2,6,10,15-tetramethyl-	1852	296	C_21_H_44_	88	0.46	< 0
	Eicosane	2009	282	C_20_H_42_	91	0.28	58.9
	Heneicosane	2109	296	C_21_H_44_	89	1.39	< 0
	5,5-Diethylheptadecane	2024	296	C_21_H_44_	87	0.25	< 0
	Tetracosane	2407	338	C_24_H_50_	91	1.20	57.2
	2-Methylhexacosane	2641	380	C_27_H_56_	88	0.12	94.8
	Octacosane	2804	394	C_28_H_58_	91	0.24	80.8
Alcohols/acids	n-Pentadecanol	1755	228	C_15_H_32_O	93	0.66	< 0
	n-Hexadecanoic acid	1968	256	C_16_H_32_O_2_	86	0.29	72.6
Aromatic compounds	(p-Hydroxyphenyl)glyoxal	1438	150	C_8_H_6_O_3_	84	0.24	54.8
	Phenol, 3,5-bis(1,1-dimethylethyl)-	1555	206	C_14_H_22_O	93	4.68	< 0
Phthalate derivatives	1,2-Benzenedicarboxylic acid, bis(2-methylpropyl) ester	1908	278	C_16_H_22_O_4_	91	0.22	61.0
	Phthalic acid, di(6-methylhept-2-yl) ester	2575	390	C_24_H_38_O_4_	87	0.55	N.A

Importantly, GC–MS peak area analysis demonstrated that many of these “recalcitrant” compounds were not completely resistant to biodegradation but instead exhibited partial removal during treatment. Therefore, this study distinguished between truly persistent compounds and slowly biodegradable compounds that underwent incomplete degradation. For example, several hydrocarbons showed substantial but incomplete removal, including nonane, 5-butyl- (81.9%), nonadecane (57.5%), eicosane (58.9%), tetracosane (57.2%), 2-methylhexacosane (94.8%), and octacosane (80.8%). Similarly, n-hexadecanoic acid showed 72.6% removal, while aromatic compounds such as (p-hydroxyphenyl)glyoxal and 1,2-benzenedicarboxylic acid, bis(2-methylpropyl) ester exhibited partial biodegradation efficiencies of 54.8% and 61.0%, respectively.

These results highlight the complexity of low-MW SMP biodegradation in MBR systems. Linear and less structurally complex hydrocarbons generally showed higher removal efficiencies, likely because their molecular structure allows easier enzymatic attack and bond cleavage. In contrast, branched hydrocarbons and aromatic compounds were more persistent due to steric hindrance and the greater stability of aromatic ring structures. Nevertheless, the partial removal of several aromatic compounds suggests that sequential anoxic-aerobic treatment promoted transformation and degradation of otherwise refractory compounds. Under anoxic conditions, complex compounds may first undergo partial transformation or cleavage into intermediate products that become more biodegradable during subsequent aerobic treatment.

The present study also demonstrates the usefulness of GC–MS for distinguishing between biodegradable, slowly biodegradable, and recalcitrant SMP fractions at the molecular level. While many previous studies have focused mainly on bulk SMP parameters such as proteins, polysaccharides, or dissolved organic carbon, relatively few have tracked the fate of individual low-MW SMP compounds across different MBR compartments. The results therefore provide new insights into the persistence and transformation behavior of specific SMP compounds during biological wastewater treatment.

Overall, the results indicate that although many low-MW SMPs generated during biological treatment were biodegradable or removable, a fraction of slowly biodegradable and recalcitrant compounds persisted in the permeate. These residual compounds may contribute to effluent dissolved organic matter and potentially influence downstream water quality, membrane fouling, and the environmental release of biologically derived organic micropollutants.

### The impact of ZnO NPs on low-MW (< 580 Da) SMPs

ZnO NP exposure substantially altered the composition and diversity of low-MW SMPs in the MBR system. The total number of GC–MS peaks detected in the aerobic compartment increased from 186 in the control reactor (MBR-C) to 225 in the ZnO NP-exposed reactor (MBR-T) (Fig. 4), indicating enhanced production and transformation of soluble organic compounds under nanoparticle stress. Among these peaks, 81 compounds in the aerobic MBR-T showed spectral match qualities greater than 80%. Alkanes remained the dominant compound class, although their relative abundance decreased compared with the control reactor, while alcohols, aromatics, and esters increased markedly (Fig. 5). In particular, the relative occurrence of alcohols increased from 14 to 23%, aromatics from 6 to 10%, and esters from 6 to 9%, whereas alkanes and alkenes generally decreased.

These compositional changes suggest that ZnO NP exposure promoted stress-induced transformation of organic matter and altered microbial metabolic pathways. Oxidative stress and membrane damage caused by ZnO NPs likely enhanced biomass turnover and partial cell lysis, releasing intracellular lipids, proteins, and other biomolecules into the bulk liquid. Subsequent degradation and transformation of these materials may explain the increased occurrence of alcohols and esters derived from lipid metabolism, as well as aromatic compounds associated with protein and amino acid degradation pathways. Microbial adaptation to nanoparticle stress may have altered membrane turnover and enzymatic activity, further contributing to the accumulation of fatty acid derivatives and secondary metabolites.

The increase in aromatic compounds also suggests the formation of partially degraded or transformed intermediates under inhibited biodegradation conditions. Some compounds were likely generated through the biological or abiotic transformation of pre-existing SMPs rather than direct microbial synthesis. Therefore, the observed shifts in SMP composition reflect combined effects of oxidative stress, altered metabolic activity, biomass decay, and transformation of dissolved organic matter under ZnO NP exposure.

Overall, Figs. 4 and 5 demonstrate that prolonged ZnO NP exposure increased both the diversity and complexity of low-MW SMPs and shifted the SMP composition toward more alcohol-, ester-, and aromatic-rich fractions. These changes indicate substantial modification of organic matter transformation pathways within the MBR system under nanoparticle stress conditions.

Supplementary Tables S5–S7 summarize the low-MW SMPs identified in the anoxic, aerobic, and permeate compartments of the ZnO NP-exposed reactor (MBR-T). Comparison with the control reactor revealed substantial changes in SMP composition under nanoparticle exposure. In the aerobic compartment, 46 compounds identified in MBR-T were also detected in the aerobic stage of MBR-C, representing approximately 57% of the identified compounds (Supplementary Tables S3 and S6). This indicates that while a significant fraction of the SMPs remained common to both systems, ZnO NP exposure also promoted the formation or accumulation of numerous additional compounds not detected in the control reactor.

Even greater differences were observed in the permeate composition. Among the 50 compounds identified with spectral match qualities greater than 80% in the MBR-T permeate, only 18 compounds (36%) were also present in the MBR-C permeate (Supplementary Tables S4 and S7). These results demonstrate that ZnO NP exposure substantially modified the composition of residual low-MW SMPs that persisted through biological treatment and membrane filtration. The presence of many unique compounds in the MBR-T permeate suggests that nanoparticle stress altered organic matter transformation pathways and promoted the accumulation of distinct soluble metabolites and transformation products.

In this study, biodegradability was not quantified through direct kinetic measurements but was inferred qualitatively from changes in compound occurrence and relative peak area across the anoxic stage, aerobic stage, and permeate. Compounds that disappeared during treatment were considered readily biodegradable or removable, whereas compounds that persisted with partial reductions in peak area were interpreted as slowly biodegradable. Persistent compounds detected throughout the treatment process were considered recalcitrant under the operating conditions applied. This approach therefore provided an assessment of apparent biodegradability and persistence of individual low-MW SMP compounds rather than absolute degradation kinetics.

Table 3 summarizes the low-MW SMP compounds detected exclusively in the ZnO NP-exposed reactor (MBR-T) and absent from the control reactor (MBR-C). To the authors’ knowledge, this is among the first studies to characterize nanoparticle-associated low-MW SMP transformation products in MBR systems using GC–MS. A total of 23 compounds were identified solely under 50 mg L^−1^ ZnO NP exposure, including alcohols, esters, aromatics, ketones, cyclic compounds, and hydrocarbons. Representative compounds included long-chain alcohols such as undecanol-3, 11-methyldodecanol, n-tetracosanol-1, and 1-eicosanol; esters such as sulfurous acid, pentadecyl 2-propyl ester and 9-octadecenoic acid methyl ester; aromatic compounds such as phthalic acid derivatives and furanone derivatives; and hydrocarbons such as undecane, decane, and branched alkane derivatives.

**Table 3:** Low-MW SMP compounds detected exclusively in the ZnO NP-exposed reactor (MBR-T) and absent from the control reactor (MBR-C)

Compound class	Compound name	RI	MW (Da)	Formula	Similarity (%)	Relative area (%)
Hydrocarbons/alkanes	Undecane, 5-methyl-	1150	170	C_12_H_26_	90	0.13
	Decane, 5,6-dimethyl-	1086	170	C_12_H_26_	81	0.12
	Cyclopropane, 1-ethyl-2-heptyl-	1178	168	C_12_H_24_	86	0.15
	2-Bromo dodecane	1446	248	C_12_H_25_Br	86	0.32
	5-Butyl-5-ethylheptadecane	2223	324	C_23_H_48_	85	0.29
	Squalane	2619	422	C_30_H_62_	84	0.17
Alcohols/oxygenated compounds	1-Heptanol, 2-propyl-	1194	158	C_10_H_22_O	90	0.40
	5-Ethyl-3-methylhept-1-en-4-ol	1039	156	C_10_H_20_O	84	0.29
	Undecanol-3	1277	172	C_11_H_24_O	81	0.08
	11-Methyldodecanol	1492	200	C_13_H_28_O	84	0.07
	Octane, 1,1′-oxybis-	1688	242	C_16_H_34_O	81	0.10
	Tridecanol, 2-ethyl-2-methyl-	1770	242	C_16_H_34_O	85	0.26
	n-Heptadecanol-1	1954	256	C_17_H_36_O	93	0.14
	Ethanol, 2-(tetradecyloxy)-	1930	258	C_16_H_34_O_2_	80	2.98
	1-Eicosanol	2252	298	C_20_H_42_O	82	0.08
	n-Tetracosanol-1	2650	354	C_24_H_50_O	82	0.12
Ketones/esters/cyclic compounds	Cyclooctanone, 2-methyl-	1192	140	C_9_H_16_O	84	0.74
	1,5-Heptadien-4-one, 3,3,6-trimethyl-	1042	152	C_10_H_16_O	82	0.09
	Propanoic acid, 2-methyl-, 3-hydroxy-2,4,4-trimethylpentyl ester	1331	216	C_12_H_24_O_3_	81	0.03
	9-Octadecenoic acid, methyl ester, (E)-	2085	296	C_19_H_36_O_2_	81	0.14
	2(3H)-Furanone, dihydro-5-tetradecyl-	2178	282	C_18_H_34_O_2_	82	0.04
	Sulfurous acid, pentadecyl 2-propyl ester	2370	334	C_18_H_38_O_3_S	86	0.41
Aromatic/phthalate compounds	Phthalic acid, di(6-methylhept-2-yl) ester	2575	390	C_24_H_38_O_4_	90	0.89

The occurrence of these compounds suggests that ZnO NP exposure substantially altered microbial metabolism and organic matter transformation pathways. Previous studies have shown that nanoparticle-induced oxidative stress can promote membrane disruption, biomass turnover, and release of intracellular constituents into the mixed liquor (Zheng et al. 2011; Wang et al. 2020). Many of the detected alcohols, esters, and fatty acid derivatives are consistent with lipid hydrolysis and membrane degradation processes, whereas aromatic and cyclic compounds may originate from incomplete biodegradation pathways or secondary metabolic responses under stress conditions (Sheng et al. 2010; Zhang et al. 2017). Recent studies have similarly reported that nanoparticle exposure can increase the diversity and complexity of dissolved organic matter through oxidative transformation and altered microbial activity (Xu and Guo 2018; Hu et al. 2025).

The fate of these compounds during treatment varied considerably. Some compounds, including propanoic acid, 2-methyl-, 3-hydroxy-2,4,4-trimethylpentyl ester, n-tetracosanol-1, and undecanol-3, were not detected in the permeate, indicating complete biodegradation or effective retention during treatment. In contrast, several compounds persisted throughout the process and exhibited only partial biodegradation. For example, tridecanol, 2-ethyl-2-methyl- and squalane showed partial removals of 45.9% and 39.3%, respectively, while compounds such as (p-hydroxyphenyl)glyoxal and 1,2-benzenedicarboxylic acid, bis(2-methylpropyl) ester displayed similar biodegradation efficiencies in both MBR-C and MBR-T. These observations suggest that ZnO NP exposure did not uniformly inhibit biodegradation but instead affected specific compounds differently depending on their structure and transformation pathways.

Interestingly, certain compounds accumulated to higher levels under ZnO NP exposure. Tetracosane was detected at concentrations approximately three times greater in MBR-T than in the control reactor, while compounds such as diethylene glycol monododecyl ether also increased substantially. Compounds including 2-methyloctacosane and squalene disappeared completely in the control reactor but persisted in the ZnO NP-exposed system. These findings suggest that ZnO NP exposure either stimulated the production and release of specific SMPs or inhibited their biodegradation. Such accumulation may result from altered enzymatic activity, inhibition of specific degradative pathways, or increased release of intracellular materials caused by oxidative stress and cell lysis.

Phthalic acid derivatives detected in this study should be interpreted cautiously. Phthalates are widely recognized as anthropogenic contaminants associated with plastics, industrial products, and laboratory materials (Net et al. 2015; Hu et al. 2021). Although procedural blanks were used to minimize contamination, the detected phthalic acid compounds may reflect a combination of background contamination, concentration effects during treatment, release from reactor-associated materials, and biological or abiotic transformation processes under ZnO NP stress. Similar observations have been reported in previous studies investigating SMP composition in biological treatment systems (Trzcinski et al. 2016).

Overall, the results demonstrate that ZnO NP exposure increased both the diversity and persistence of low-MW SMP compounds and promoted the accumulation of several slowly biodegradable or recalcitrant constituents. However, the effects of ZnO NPs on SMP production and biodegradation were highly compound-specific, indicating that no universal relationship exists between nanoparticle exposure and SMP behavior. These findings highlight the complexity of nanoparticle-induced transformations in MBR systems and the importance of molecular-level characterization for understanding SMP dynamics under environmental stress conditions.

## Conclusions

Long-term ZnO NP exposure altered SMP characteristics in submerged anoxic-aerobic MBRs at both bulk and molecular levels. The integrated analytical results indicate that nanoparticle stress affected extracellular polymer production, SMP molecular-weight distribution, fluorescent organic-matter fractions, and the composition of extractable low-MW compounds. Most importantly, ZnO NP exposure promoted the formation and persistence of a more chemically diverse and relatively recalcitrant SMP fraction.

The persistence of long-chain hydrocarbons, aromatic derivatives, and other slowly biodegradable compounds in the permeate indicates that ZnO NPs deteriorate MBR effluent quality by increasing the residual organic burden. Therefore, assessment of nanoparticle impacts should consider not only total SMP concentration but also SMP composition, persistence, and treatability. Molecular-weight profiling, fluorescence characterization, and targeted analysis of persistent low-MW compounds may provide useful complementary indicators of nanoparticle-associated stress and potential effluent-quality deterioration.

Future studies should evaluate environmentally relevant ZnO NP concentrations in real wastewater, separate particulate and ionic effects, and directly relate SMP transformation to membrane fouling, effluent biodegradability, and toxicity using membrane autopsy and high-resolution non-target mass spectrometry.

## Supplementary Information

Below is the link to the electronic supplementary material.

ESM 1 (DOCX 1.18 MB)

ESM 2 (DOCX 99.2 KB)

## Data Availability

Data can be provided on request.
