# Supplementary material for: Long-term effects of zinc oxide nanoparticles on soluble microbial product composition and transformation in submerged membrane bioreactors
Source: Environ Sci Pollut Res Int. 2026 Aug 14;33(26):13459–74. doi: 10.1007/s11356-026-38139-3 (PMC13503389; doi:10.1007/s11356-026-38139-3)
Supplement: Supplementary file 1 [file 11356_2026_38139_MOESM1_ESM.docx]

Fig. S1. Schematic diagram of the MBR systems

|  |  |  |
| --- | --- | --- |

Fig. S2. Transmission electron microscopy (TEM) images of ZnO NPs in solution. The particle sizes ranged from 15-47 nm, with a mean size of 33± 8 nm (n=107)
