# Supplementary material for: Long-term effects of zinc oxide nanoparticles on soluble microbial product composition and transformation in submerged membrane bioreactors
Source: Environ Sci Pollut Res Int. 2026 Aug 14;33(26):13459–74. doi: 10.1007/s11356-026-38139-3 (PMC13503389; doi:10.1007/s11356-026-38139-3)
Supplement: Supplementary file 2 [file 11356_2026_38139_MOESM2_ESM.docx]

Table S1. Alkane standards for the identification of low-MW SMPs

| Compound name | Formula | Molecular weight (Da) | Retention time (min) | Retention index |
| --- | --- | --- | --- | --- |
| Decane | C_10_H_22_ | 142 | 12.15 | 1015 |
| Dodecane | C_12_H_26_ | 170 | 17.16 | 1214 |
| Tetradecane | C_14_H_30_ | 198 | 21.96 | 1413 |
| Hexadecane | C_16_H_34_ | 226 | 25.70 | 1612 |
| Octadecane | C_18_H_38_ | 254 | 29.03 | 1810 |
| Eicosane | C_20_H_42_ | 282 | 32.04 | 2009 |
| Docosane | C_22_H_46_ | 310 | 34.78 | 2208 |
| Tetracosane | C_24_H_50_ | 338 | 37.30 | 2427 |
| Hexacosane | C_26_H_54_ | 366 | 39.62 | 2606 |
| Octacosane | C_28_H_58_ | 394 | 41.77 | 2804 |
| Triacontane | C_30_H_62_ | 422 | 43.78 | 3003 |
| Dotriacontane | C_32_H_66_ | 450 | 45.66 | 3202 |
| Tetratriacontane | C_34_H_70_ | 478 | 47.51 | 3401 |
| Hexatriacontane | C_36_H_74_ | 506 | 49.72 | 3600 |
| Octatriacontane | C_38_H_78_ | 534 | 52.59 | 3800 |
| Tetracontane | C_40_H_82_ | 562 | 56.45 | 3997 |

Table S2. Compounds detected by GC-MS in the anoxic compartment of MBR-C (% match > 80%)

| **Similarity** | **Retention Index** | **Compound Name** | **Molecular Weight (Da)** | **Chemical Formula** | **Area** | **Percentage**  **(%)** |
| --- | --- | --- | --- | --- | --- | --- |
| 92 | 1158 | 3-Undecene, 6-methyl-, (E)- | 168 | C_12_H_24_ | 1,935,868 | 2.09 |
| 81 | 1126 | 1,2,3-Trimethylpiperidin-4-one | 141 | C_8_H_15_NO | 1,575,321 | 1.7 |
| 91 | 1066 | 1-Hexanol, 5-methyl-2-(1-methylethyl)- | 158 | C_10_H_22_O | 83,337 | 0.09 |
| 82 | 1197 | 1,3-Cyclohexanedione, 5,5-dimethyl- | 140 | C_8_H_12_O_2_ | 1,731,071 | 1.87 |
| 92 | 1159 | 1-Nonanol | 144 | C_9_H_20_O | 358,542 | 0.39 |
| 89 | 1059 | 1-Octanol | 130 | C_8_H_18_O | 99,534 | 0.11 |
| 81 | 1131 | Cyclooctanone | 126 | C_8_H_14_O | 278,895 | 0.3 |
| 82 | 1091 | Octane, 1-ethoxy- | 158 | C_10_H_22_O | 700,445 | 0.76 |
| 89 | 1263 | Benzenemethanol, 3,5-dimethyl- | 136 | C_9_H_12_O | 129,890 | 0.14 |
| 86 | 1228 | Phenol, 2-(1,1-dimethylethyl)- | 150 | C_10_H_14_O | 89,494 | 0.1 |
| 92 | 1249 | Nonane, 5-butyl- | 184 | C_13_H_28_ | 233,172 | 0.25 |
| 94 | 1228 | Phenol, m-tert-butyl- | 150 | C_10_H_14_O | 610,261 | 0.66 |
| 92 | 1285 | Dodecane, 4,6-dimethyl- | 198 | C_14_H_30_ | 211,562 | 0.23 |
| 88 | 1347 | Propanoic acid, 2-methyl-, 2,2-dimethyl-1-(2-hydroxy-1-methylethyl)propyl ester | 216 | C_12_H_24_O_3_ | 128,705 | 0.14 |
| 83 | 1376 | 3-Dodecanol | 186 | C_12_H_26_O | 901,83 | 0.1 |
| 95 | 1304 | 1-Tridecene | 182 | C_13_H_26_ | 2,658,690 | 2.88 |
| 94 | 1249 | Decane, 5-propyl- | 184 | C_13_H_28_ | 197,377 | 0.21 |
| 91 | 1421 | 7-Tetradecene, (E)- | 196 | C_14_H_28_ | 100,685 | 0.11 |
| 93 | 1421 | 3-Tetradecene, (E)- | 196 | C_14_H_28_ | 173,925 | 0.19 |
| 85 | 1438 | (p-Hydroxyphenyl)glyoxal | 150 | C_8_H_6_O_3_ | 534,930 | 0.58 |
| 94 | 1413 | Tetradecane | 198 | C_14_H_30_ | 111,871 | 0.12 |
| 94 | 1555 | Phenol, 2,4-bis(1,1-dimethylethyl)- | 206 | C_14_H_22_O | 10,494,150 | 11.37 |
| 90 | 1479 | 2-Methyl-1-tetradecene | 210 | C_15_H_30_ | 98,082 | 0.11 |
| 96 | 1514 | Trifluoroacetic acid,n-tridecyl ester | 296 | C_15_H_27_F_3_O_2_ | 2,859,018 | 3.09 |
| 95 | 1512 | Pentadecane | 212 | C_15_H_32_ | 657,410 | 0.71 |
| 87 | 1605 | Pentanoic acid, 2,2,4-trimethyl-3-carboxyisopropyl, isobutyl ester | 286 | C_16_H_30_O_4_ | 678,346 | 0.73 |
| 84 | 1602 | Cetene | 224 | C_16_H_32_ | 119,881 | 0.13 |
| 85 | 1609 | 2(3H)-Benzothiazolethione, 3-methyl- | 181 | C_8_H_7_NS_2_ | 92,605 | 0.1 |
| 88 | 1603 | Benzophenone | 182 | C_13_H_10_O | 192,979 | 0.21 |
| 95 | 1656 | 1-Tetradecanol | 214 | C_14_H_30_O | 555,225 | 0.6 |
| 95 | 1711 | Heptadecane | 240 | C_17_H_36_ | 1,359,290 | 1.47 |
| 94 | 1740 | Methanone, (1-hydroxycyclohexyl)phenyl- | 204 | C_13_H_16_O_2_ | 590,109 | 0.64 |
| 94 | 1731 | Ethanol, 2-(dodecyloxy)- | 230 | C_14_H_30_O_2_ | 1,043,116 | 1.13 |
| 89 | 1678 | 2-Methyl-1-hexadecene | 238 | C_17_H_34_ | 203,044 | 0.22 |
| 86 | 1733 | Heptafluorobutyric acid, hexadecyl ester | 438 | C_20_H_33_F_7_O_2_ | 167,471 | 0.18 |
| 85 | 1716 | Methanone, (3-methylphenyl)phenyl- | 196 | C_14_H_12_O | 216,086 | 0.23 |
| 96 | 1755 | n-Pentadecanol | 228 | C_15_H_32_O | 2465,930 | 2.67 |
| 80 | 1852 | Heptadecane, 2,6,10,15-tetramethyl- | 296 | C_21_H_44_ | 313,562 | 0.34 |
| 82 | 1805 | p-Toluic acid, 2-ethylhexyl ester | 248 | C_16_H_24_O_2_ | 184,218 | 0.2 |
| 86 | 1833 | Heptadecyl heptafluorobutyrate | 452 | C_21_H_35_F_7_O_2_ | 134,089 | 0.15 |
| 83 | 1846 | Octadecane, 2-methyl- | 268 | C_19_H_40_ | 42,952 | 0.05 |
| 96 | 1908 | 1,2-Benzenedicarboxylic acid, bis(2-methylpropyl) ester | 278 | C1_6_H_22_O_4_ | 1,303,798 | 1.41 |
| 92 | 1910 | Nonadecane | 268 | C_19_H_40_ | 318,723 | 0.34 |
| 95 | 1878 | Hexadecanoic acid, methyl ester | 270 | C_17_H_34_O_2_ | 2,358,419 | 2.55 |
| 86 | 1945 | 10-Methylnonadecane | 282 | C_20_H_42_ | 142,831 | 0.15 |
| 94 | 1968 | n-Hexadecanoic acid | 256 | C_16_H_32_O_2_ | 1,299,111 | 1.41 |
| 97 | 1900 | 1-Nonadecene | 266 | C_19_H_38_ | 1,634,624 | 1.77 |
| 84 | 1890 | Methoxyacetic acid, 2-tetradecyl ester | 286 | C_17_H_34_O_3_ | 1,669,272 | 1.81 |
| 91 | 2024 | 5,5-Diethylheptadecane | 296 | C_21_H_44_ | 282,234 | 0.31 |
| 83 | 2013 | Isopropyl palmitate | 298 | C_19_H_38_O_2_ | 112,346 | 0.12 |
| 91 | 2024 | 3,3-Diethylheptadecane | 296 | C_21_H_44_ | 127,769 | 0.14 |
| 91 | 2011 | Octadecyl trifluoroacetate | 366 | C_20_H_37_F_3_O_2_ | 417,642 | 0.45 |
| 97 | 2153 | n-Nonadecanol-1 | 284 | C_19_H_40_O | 489,892 | 0.53 |
| 90 | 2109 | Heneicosane | 296 | C_21_H_44_ | 145,297 | 0.16 |
| 85 | 2085 | 9-Octadecenoic acid (Z)-, methyl ester | 296 | C_19_H_36_O_2_ | 171,366 | 0.19 |
| 93 | 2077 | Methyl stearate | 298 | C_19_H3_8_O_2_ | 68,136 | 0.07 |
| 88 | 2188 | 1-Dodecanol, 2-octyl- | 298 | C_20_H_42_O | 138,475 | 0.15 |
| 90 | 2167 | Octadecanoic acid | 284 | C_18_H_36_O_2_ | 151,463 | 0.16 |
| 89 | 2045 | Pentadecane, 8-hexyl- | 296 | C_21_H_44_ | 272,521 | 0.29 |
| 85 | 2143 | 2-Butenedioic acid (Z)-, monododecyl ester | 284 | C_16_H_28_O_4_ | 224,838 | 0.24 |
| 93 | 2009 | Eicosane | 282 | C_20_H_42_ | 800,040 | 0.87 |
| 91 | 2006 | Diethylene glycol monododecyl ether | 274 | C_16_H_34_O_3_ | 370,695 | 0.4 |
| 87 | 2477 | Heneicosane, 11-(1-ethylpropyl)- | 366 | C_26_H_54_ | 285,968 | 0.31 |
| 80 | 2354 | Heptaethylene glycol | 326 | C_14_H_30_O_8_ | 275,366 | 0.3 |
| 90 | 2351 | 1-Heneicosanol | 312 | C_21_H_44_O | 669,548 | 0.72 |
| 81 | 2506 | 10-Cyclohexylnonadecane | 350 | C_25_H_50_ | 511,273 | 0.55 |
| 87 | 2328 | Ethanol, 2-(octadecyloxy)- | 314 | C_20_H_42_O_2_ | 65,417 | 0.07 |
| 88 | 2407 | Tetracosane | 338 | C_24_H_50_ | 32,944 | 0.04 |
| 90 | 2451 | Behenic alcohol | 326 | C_22_H_46_O | 293,117 | 0.32 |
| 82 | 2498 | Hexadecanoic acid, 2-hydroxy-1-(hydroxymethyl)ethyl ester | 330 | C_19_H_38_O_4_ | 78,120 | 0.08 |
| 88 | 2442 | 2-methyltetracosane | 352 | C_25_H_52_ | 38,765 | 0.04 |
| 87 | 2556 | Tetraethylene glycol monododecyl ether | 362 | C_20_H_42_O_5_ | 82,974 | 0.09 |
| 90 | 2575 | Phthalic acid, di(6-methylhept-2-yl) ester | 390 | C_24_H_38_O_4_ | 416,261 | 0.45 |
| 84 | 2740 | Docosane, 7-hexyl- | 394 | C_28_H_58_ | 324,439 | 0.35 |
| 91 | 2641 | 2-methylhexacosane | 380 | C_27_H_56_ | 434,289 | 0.47 |
| 85 | 2668 | Sulfurous acid, octadecyl 2-propyl ester | 376 | C_21_H_44_O_3_S | 125,022 | 0.14 |
| 90 | 2804 | Octacosane | 394 | C_28_H_58_ | 154,886 | 0.17 |
| 88 | 2840 | 2-methyloctacosane | 408 | C_29_H_60_ | 56,740 | 0.06 |
| 78 | 2830 | Pentaethylene glycol monododecyl ether | 406 | C_22_H_46_O_6_ | 97,017 | 0.1 |
| 86 | 2831 | Sulfurous acid, butyl octadecyl ester | 390 | C_22_H_46_O_3_S | 146,406 | 0.16 |
| 83 | 3138 | Docosane, 11-decyl- | 450 | C_32_H_66_ | 92,877 | 0.1 |
| 89 | 3047 | Octacosanol | 410 | C_28_H_58_O | 221,360 | 0.24 |
| 87 | 3103 | Hentriacontane | 436 | C_31_H_64_ | 305,202 | 0.33 |
| 85 | 3401 | Tetratriacontane | 478 | C_34_H_70_ | 152,634 | 0.17 |

Table S3. Compounds detected by GC-MS in the aerobic compartment of MBR-C (% match > 80%).

| **Similarity** | **Retention Index** | **Compound Name** | **Molecular Weight**  **(Da)** | **Chemical Formula** | **Area** | **Area**  **Percentage**  **(%)** |
| --- | --- | --- | --- | --- | --- | --- |
| 96 | 995 | 1-Hexanol, 2-ethyl- | 130 | C_8_H_18_O | 433,728 | 0.64 |
| 80 | 1070 | 2,6-Heptadien-1-ol, 2,4-dimethyl- | 140 | C_9_H_16_O | 416,966 | 0.61 |
| 89 | 1185 | Undecane, 3,7-dimethyl- | 184 | C_13_H_28_ | 530,527 | 0.78 |
| 83 | 1086 | Octane, 4,5-diethyl- | 170 | C_12_H_26_ | 171,187 | 0.25 |
| 82 | 1057 | 1-(2,4-Dimethyl-furan-3-yl)-ethanone | 138 | C_8_H_10_O_2_ | 176,280 | 0.26 |
| 93 | 1185 | Nonane, 5-(2-methylpropyl)- | 184 | C_13_H_28_ | 187,862 | 0.28 |
| 92 | 1086 | Decane, 3,7-dimethyl- | 170 | C_12_H_26_ | 176,307 | 0.26 |
| 82 | 1197 | 1,3-Cyclohexanedione, 5,5-dimethyl- | 140 | C_8_H_12_O_2_ | 282,461 | 0.42 |
| 82 | 1162 | 6-Nitrohexan-2-ol | 147 | C_6_H_13_NO_3_ | 68,831 | 0.1 |
| 88 | 1272 | Nonanoic acid | 158 | C_9_H_18_O_2_ | 94,541 | 0.14 |
| 94 | 1285 | Dodecane, 4,6-dimethyl- (*) | 198 | C_14_H_30_ | 468,387 | 0.69 |
| 92 | 1320 | Dodecane, 2,6,11-trimethyl- | 212 | C_15_H_32_ | 122,796 | 0.18 |
| 92 | 1249 | Nonane, 5-butyl- (*) | 184 | C_13_H_28_ | 428,129 | 0.63 |
| 91 | 1249 | Dodecane, 4-methyl- | 184 | C_13_H_28_ | 163,010 | 0.24 |
| 93 | 1304 | 1-Tridecene (*) | 182 | C_13_H_26_ | 73,168 | 0.11 |
| 92 | 1313 | Tridecane | 184 | C_13_H_28_ | 61,259 | 0.09 |
| 85 | 1438 | (p-Hydroxyphenyl)glyoxal (*) | 150 | C_8_H_6_O_3_ | 245,329 | 0.36 |
| 96 | 1457 | 1-Dodecanol | 186 | C_12_H_26_O | 516,224 | 0.76 |
| 95 | 1512 | Pentadecane (*) | 212 | C_15_H_32_ | 656,974 | 0.97 |
| 86 | 1519 | Tetradecane, 2,6,10-trimethyl- | 240 | C_17_H_36_ | 292,671 | 0.43 |
| 93 | 1555 | Phenol, 3,5-bis(1,1-dimethylethyl)- (*) | 206 | C_14_H_22_O | 106,580 | 0.16 |
| 90 | 1413 | Tetradecane (*) | 198 | C_14_H_30_ | 180,125 | 0.26 |
| 86 | 1448 | Tetradecane, 4-methyl- | 212 | C_15_H_32_ | 124,766 | 0.18 |
| 89 | 1430 | Decane, 1-iodo- | 268 | C_10_H_21_I | 113,093 | 0.17 |
| 95 | 1502 | 1-Pentadecene | 210 | C_15_H_30_ | 321,197 | 0.47 |
| 81 | 1432 | Propanoic acid, 2-methyl-, 2-(hydroxymethyl)-1-propylbutyl ester | 216 | C_12_H_24_O_3_ | 172,825 | 0.25 |
| 96 | 1656 | 1-Tetradecanol (*) | 214 | C_14_H_30_O | 385,933 | 0.57 |
| 83 | 1769 | 2-Propenoic acid, tridecyl ester | 254 | C_16_H_30_O_2_ | 113,403 | 0.17 |
| 95 | 1711 | Heptadecane (*) | 240 | C_17_H_36_ | 235,083 | 0.35 |
| 91 | 1746 | Heptadecane, 8-methyl- | 254 | C_18_H_38_ | 680,093 | 1 |
| 88 | 1790 | 1-Decanol, 2-hexyl- | 242 | C_16_H_34_O | 990,732 | 1.46 |
| 90 | 1753 | Hexadecane, 2,6,11,15-tetramethyl- | 282 | C_20_H_42_ | 808,673 | 1.19 |
| 84 | 1645 | 2-Bromotetradecane | 276 | C_14_H_29_Br | 82,856 | 0.12 |
| 85 | 1627 | 5,5-Diethyltridecane | 240 | C_17_H_36_ | 151,000 | 0.22 |
| 94 | 1755 | n-Pentadecanol (*) | 228 | C_15_H_32_O | 101,516 | 0.15 |
| 94 | 1612 | Hexadecane | 226 | C_16_H_34_ | 478,795 | 0.7 |
| 93 | 1908 | 1,2-Benzenedicarboxylic acid, bis(2-methylpropyl) ester (*) | 278 | C_16_H_22_O_4_ | 260,483 | 0.38 |
| 86 | 1748 | 2-Hexadecanone | 240 | C_16_H_32_O | 62,309 | 0.09 |
| 80 | 1945 | 10-Methylnonadecane (*) | 282 | C_20_H_42_ | 1,792,226 | 2.63 |
| 92 | 1852 | Heptadecane, 2,6,10,15-tetramethyl- (*) | 296 | C_21_H_44_ | 162,501 | 0.24 |
| 91 | 1968 | n-Hexadecanoic acid (*) | 256 | C_16_H_32_O_2_ | 487,219 | 0.72 |
| 88 | 1825 | 5,5-Diethylpentadecane | 268 | C_19_H_40_ | 1,136,186 | 1.67 |
| 86 | 1810 | Octadecane | 254 | C_18_H_38_ | 329,943 | 0.48 |
| 95 | 1900 | 1-Nonadecene (*) | 266 | C_19_H_38_ | 516,435 | 0.76 |
| 84 | 1910 | Nonadecane (*) | 268 | C_19_H_40_ | 1,769,629 | 2.6 |
| 90 | 2009 | Eicosane (*) | 282 | C_20_H_42_ | 316,505 | 0.47 |
| 90 | 2109 | Heneicosane (*) | 296 | C_21_H_44_ | 191,241 | 0.53 |
| 84 | 2013 | Isopropyl palmitate (*) | 298 | C1_9_H_38_O_2_ | 126,313 | 0.19 |
| 87 | 2024 | 5,5-Diethylheptadecane (*) | 296 | C_21_H_44_ | 83,296 | 0.12 |
| 91 | 2153 | n-Nonadecanol-1 (*) | 284 | C_19_H_40_O | 59,878 | 0.09 |
| 82 | 2198 | 1-Docosene | 308 | C_22_H_44_ | 42,626 | 0.06 |
| 90 | 2167 | Octadecanoic acid | 284 | C_18_H_36_O_2_ | 170,133 | 0.25 |
| 88 | 2188 | 1-Dodecanol, 2-octyl- (*) | 298 | C_20_H_42_O | 227,369 | 0.33 |
| 88 | 2026 | Hexadecane, 1-iodo- | 352 | C_16_H_33_I | 1,045,520 | 1.54 |
| 92 | 2045 | Pentadecane, 8-hexyl- (*) | 296 | C_21_H_44_ | 1,016,446 | 1.49 |
| 85 | 2129 | Ethanol, 2-(hexadecyloxy)- | 286 | C_18_H_38_O_2_ | 427,543 | 0.63 |
| 94 | 2281 | Triethylene glycol monododecyl ether | 318 | C_18_H_38_O_4_ | 666,817 | 0.98 |
| 92 | 2407 | Tetracosane (*) | 338 | C_24_H_50_ | 1,296,751 | 1.91 |
| 86 | 2556 | Tetraethylene glycol monododecyl ether (*) | 362 | C_20_H_42_O_5_ | 270,984 | 0.4 |
| 97 | 2704 | Bis(2-ethylhexyl) phthalate | 390 | C_24_H_38_O_4_ | 10,552,436 | 15.49 |
| 86 | 2442 | 2-methyltetracosane (*) | 352 | C_25_H_52_ | 335,439 | 0.49 |
| 85 | 1753 | Hexadecane, 2,6,10,14-tetramethyl- | 282 | C_20_H_42_ | 76,315 | 0.11 |
| 93 | 2641 | 2-methylhexacosane (*) | 380 | C_27_H_56_ | 1,098,519 | 1.61 |
| 85 | 2840 | 2-methyloctacosane (*) | 408 | C_29_H_60_ | 479,910 | 0.71 |
| 83 | 2914 | Squalene | 410 | C_30_H_50_ | 87,809 | 0.13 |
| 90 | 2804 | Octacosane (*) | 394 | C_28_H_58_ | 579,294 | 0.85 |
| 83 | 3165 | Sulfurous acid, 2-ethylhexyl octadecyl ester | 446 | C_26_H_54_O_3_S | 132,135 | 0.19 |
| 88 | 3047 | Octacosanol (*) | 410 | C_28_H_58_O | 108,859 | 0.16 |
| 89 | 3103 | Hentriacontane | 436 | C_31_H_64_ | 611,641 | 0.9 |
| 84 | 3500 | Pentatriacontane | 492 | C_35_H_72_ | 100,791 | 0.15 |
| 90 | 3401 | Tetratriacontane (*) | 478 | C_34_H_70_ | 342,866 | 0.5 |
| 88 | 3600 | Hexatriacontane | 506 | C_36_H_74_ | 177,538 | 0.26 |

Note:

1. (*) represents the compound that was also detected in the anoxic compartment of MBR-C.

Table S4. Compounds detected by GC-MS in the MBR-C permeate (% match > 80%).

| **Similarity** | **Retention Index** | **Compound Name** | **Molecular Weight**  **(Da)** | **Chemical Formula** | **Area** | **Area**  **percentage**  **(%)** | **Removal**  **(%)** |
| --- | --- | --- | --- | --- | --- | --- | --- |
| 82 | 1057 | 1-(2,4-Dimethyl-furan-3-yl)-ethanone | 138 | C_8_H_10_O_2_ | 295,223 | 0.64 | N.A. |
| 92 | 1159 | 1-Nonanol | 144 | C_9_H_20_O | 324,857 | 0.7 | N.A. |
| 91 | 1204 | 1-Dodecene | 168 | C_12_H_24_ | 265,549 | 0.57 | N.A. |
| 94 | 1285 | Dodecane, 4,6-dimethyl- (**) | 198 | C_14_H_30_ | 439,890 | 0.95 | 6.1 |
| 91 | 1249 | Nonane, 5-butyl- (**) | 184 | C_13_H_28_ | 77,569 | 0.17 | 81.9 |
| 93 | 1320 | Dodecane, 2,6,11-trimethyl- (*) | 212 | C1_5_H_32_ | 403,044 | 0.87 | <0 |
| 91 | 1249 | Dodecane, 4-methyl- (*) | 184 | C1_3_H_28_ | 146,426 | 0.32 | 10.2 |
| 95 | 1304 | Tridecane (**) | 182 | C_13_H_26_ | 301,871 | 0.65 | <0 |
| 93 | 1313 | n-Tridecan-1-ol | 184 | C_13_H_28_ | 82,109 | 0.18 | N.A. |
| 84 | 1438 | (p-Hydroxyphenyl)glyoxal (**) | 150 | C_8_H_6_O_3_ | 110,883 | 0.24 | 54.8 |
| 90 | 1512 | Pentadecane (**) | 212 | C_15_H_32_ | 623,007 | 1.34 | 5.1 |
| 85 | 1519 | Tetradecane, 2,6,10-trimethyl- (*) | 240 | C_17_H_36_ | 179,534 | 0.39 | 38.7 |
| 93 | 1555 | Phenol, 3,5-bis(1,1-dimethylethyl)- (**) | 206 | C_14_H_22_O | 2,168,254 | 4.68 | <0 |
| 84 | 1489 | Hexyl octyl ether | 214 | C_14_H_30_O | 115,988 | 0.25 | N.A. |
| 90 | 1430 | Decane, 1-iodo- (*) | 268 | C_10_H_21_I | 85,105 | 0.18 | 24.8 |
| 95 | 1502 | 1-Pentadecene | 210 | C_15_H_30_ | 286,238 | 0.62 | N.A. |
| 95 | 1413 | Tetradecane (**) | 198 | C_14_H_30_ | 241,853 | 0.52 | <0 |
| 83 | 1605 | Propanoic acid, 2-methyl-, 1-(1,1-dimethylethyl)-2-methyl-1,3-propanediyl ester | 286 | C_16_H_30_O_4_ | 54,067 | 0.12 | N.A. |
| 91 | 1746 | Heptadecane, 8-methyl- (*) | 254 | C_18_H_38_ | 624,428 | 1.35 | 8.2 |
| 93 | 1731 | Ethanol, 2-(dodecyloxy)- | 230 | C_14_H_30_O_2_ | 279,393 | 0.6 | N.A. |
| 91 | 1711 | Heptadecane (**) | 240 | C_17_H_36_ | 655,494 | 1.41 | <0 |
| 83 | 1753 | Hexadecane, 2,6,11,15-tetramethyl- (*) | 282 | C_20_H_42_ | 31,685 | 0.07 | 96.1 |
| 86 | 1628 | Dodecane, 1-iodo- | 296 | C_12_H_25_I | 76,292 | 0.16 | N.A. |
| 93 | 1755 | n-Pentadecanol (**) | 228 | C_15_H_32_O | 304,786 | 0.66 | <0 |
| 95 | 1612 | Hexadecane (*) | 226 | C_16_H_34_ | 429,407 | 0.93 | 10.4 |
| 87 | 1645 | 2-Bromotetradecane (*) | 276 | C_14_H_29_Br | 129,649 | 0.15 | <0 |
| 91 | 1908 | 1,2-Benzenedicarboxylic acid, bis(2-methylpropyl) ester (**) | 278 | C_16_H_22_O_4_ | 101,649 | 0.22 | 61.0 |
| 87 | 1847 | 2-Heptadecanone | 254 | C_17_H_34_O | 35,495 | 0.08 | N.A. |
| 83 | 1945 | 10-Methylnonadecane (**) | 282 | C_20_H_42_ | 1,041,897 | 2.25 | 41.9 |
| 86 | 1968 | n-Hexadecanoic acid (**) | 256 | C_16_H_32_O_2_ | 133,235 | 0.29 | 72.6 |
| 88 | 1825 | 5,5-Diethylpentadecane (*) | 268 | C_19_H_4_0 | 855,059 | 1.84 | 24.7 |
| 88 | 1810 | Octadecane (*) | 254 | C_18_H_38_ | 225,776 | 0.49 | 31.6 |
| 93 | 1900 | 1-Nonadecene (**) | 266 | C_19_H_38_ | 322,142 | 0.69 | 37.6 |
| 90 | 1910 | Nonadecane (**) | 268 | C_19_H_40_ | 752,639 | 1.62 | 57.5 |
| 88 | 1852 | Heptadecane, 2,6,10,15-tetramethyl- (**) | 296 | C_21_H_44_ | 211,969 | 0.46 | <0 |
| 91 | 2009 | Eicosane (**) | 282 | C_20_H_42_ | 130,080 | 0.28 | 58.9 |
| 89 | 2109 | Heneicosane (**) | 296 | C_21_H_44_ | 644,467 | 1.39 | <0 |
| 87 | 2024 | 5,5-Diethylheptadecane (**) | 296 | C_21_H_44_ | 114,816 | 0.25 | <0 |
| 88 | 2026 | Hexadecane, 1-iodo- (*) | 352 | C_16_H_33_I | 847,993 | 1.83 | 18.9 |
| 84 | 2089 | Methoxyacetic acid, 4-hexadecyl ester | 314 | C_19_H_38_O_3_ | 163,657 | 0.35 | N.A. |
| 94 | 2281 | Triethylene glycol monododecyl ether (*) | 318 | C_18_H_38_O_4_ | 203,596 | 0.44 | 69.5 |
| 87 | 2575 | Phthalic acid, di(6-methylhept-2-yl) ester | 390 | C_24_H_38_O_4_ | 253,544 | 0.55 | N.A. |
| 91 | 2407 | Tetracosane (**) | 338 | C_24_H_50_ | 554,768 | 1.2 | 57.2 |
| 88 | 2641 | 2-methylhexacosane (**) | 380 | C_27_H_56_ | 57,605 | 0.12 | 94.8 |
| 91 | 2804 | Octacosane (**) | 394 | C_28_H_58_ | 111,367 | 0.24 | 80.8 |

Note:

1. (*) represents the compound that was also detected in the aerobic compartment of MBR-C.
2. (**) represents the compound that was also detected in the anoxic and aerobic compartments of MBR-C.

Table S5. Compounds detected by GC-MS in the anoxic compartment of MBR-T (% match > 80%)

| **Similarity** | **Retention Index** | **Compound Name** | **Molecular Weight (Da)** | **Chemical Formula** | **Area** | **Percentage**  **(%)** |
| --- | --- | --- | --- | --- | --- | --- |
| 86 | 789 | 2-Hexanone, 5-methyl- | 114 | C_7_H_14_O | 493,913 | 0.46 |
| 92 | 1158 | 3-Undecene, 6-methyl-, (E)- | 168 | C_12_H_24_ | 3,003,629 | 2.78 |
| 82 | 1112 | 1-Undecyne | 152 | C_11_H_20_ | 512,276 | 0.47 |
| 91 | 1086 | Decane, 3,7-dimethyl- | 170 | C_12_H_26_ | 138,017 | 0.13 |
| 84 | 1140 | 1-Undecene, 7-methyl- | 168 | C_12_H_24_ | 79,628 | 0.07 |
| 83 | 1057 | 1-(2,4-Dimethyl-furan-3-yl)-ethanone | 138 | C_8_H_10_O_2_ | 95,494 | 0.09 |
| 90 | 1194 | 1-Heptanol, 2-propyl- | 158 | C_10_H_22_O | 248,383 | 0.23 |
| 92 | 1185 | Nonane, 5-(2-methylpropyl)- | 184 | C_13_H_28_ | 305,242 | 0.28 |
| 89 | 1156 | Decane, 2,3,5,8-tetramethyl- | 198 | C_14_H_30_ | 414,471 | 0.38 |
| 81 | 1079 | Cyclohexane, butyl- | 140 | C_10_H_20_ | 815,944 | 0.75 |
| 86 | 1178 | 5-Decanol | 158 | C_10_H_22_O | 375,127 | 0.35 |
| 92 | 1117 | Cyclopropane, octyl- | 154 | C_11_H_22_ | 202,445 | 0.19 |
| 89 | 1094 | (S)-(+)-6-Methyl-1-octanol | 144 | C_9_H_20_O | 88,738 | 0.08 |
| 81 | 1091 | 3-Methyl-2-butenoic acid, cyclobutyl ester | 154 | C_9_H_14_O_2_ | 80,869 | 0.07 |
| 83 | 1039 | 5-Ethyl-3-methylhept-1-en-4-ol | 156 | C_10_H_20_O | 178,842 | 0.17 |
| 94 | 1285 | Dodecane, 4,6-dimethyl- | 198 | C_14_H_30_ | 456,607 | 0.42 |
| 90 | 1185 | Nonane, 3-methyl-5-propyl- | 184 | C_13_H_28_ | 60,029 | 0.06 |
| 87 | 1249 | Dodecane, 4-methyl- | 184 | C_13_H_28_ | 143,350 | 0.13 |
| 84 | 1229 | Nonane, 5-methyl-5-propyl- | 184 | C_13_H_28_ | 70,668 | 0.07 |
| 93 | 1320 | Dodecane, 2,6,11-trimethyl- | 212 | C_15_H_32_ | 408,178 | 0.38 |
| 82 | 1391 | 2-Butenoic acid, 2-propenylidene ester | 210 | C_11_H_14_O_4_ | 219,040 | 0.2 |
| 91 | 1249 | Dodecane, 2-methyl- | 184 | C_13_H_28_ | 175,338 | 0.16 |
| 91 | 1304 | 1-Tridecene | 182 | C_13_H_26_ | 401,389 | 0.37 |
| 93 | 1313 | Tridecane | 184 | C_13_H_28_ | 91,479 | 0.08 |
| 86 | 1438 | (p-Hydroxyphenyl)glyoxal | 150 | C_8_H_6_O_3_ | 435,358 | 0.4 |
| 96 | 1457 | 1-Dodecanol | 186 | C_12_H_26_O | 922,996 | 0.85 |
| 80 | 1469 | 2-Pentanone, 4-(1,3,3-trimethyl-7-oxabicyclo[4.1.0]hept-2-yl)- | 224 | C_14_H_24_O_2_ | 338,690 | 0.31 |
| 94 | 1555 | Phenol, 2,4-bis(1,1-dimethylethyl)- | 206 | C_14_H_22_O | 5,472,394 | 5.11 |
| 91 | 1512 | Pentadecane | 212 | C_15_H_32_ | 702,824 | 0.65 |
| 87 | 1446 | 2-Bromo dodecane | 248 | C_12_H_25_Br | 300,320 | 0.28 |
| 81 | 1492 | 1-Dodecanol, 2-methyl-, (S)- | 200 | C_13_H_28_O | 63,667 | 0.06 |
| 86 | 1519 | Tetradecane, 2,6,10-trimethyl- | 240 | C_17_H_36_ | 170,976 | 0.16 |
| 89 | 1430 | Decane, 1-iodo- | 268 | C_10_H_21_I | 117,878 | 0.11 |
| 95 | 1502 | 1-Pentadecene | 210 | C_15_H_30_ | 791,511 | 0.73 |
| 95 | 1413 | Tetradecane | 198 | C_14_H_30_ | 424,648 | 0.39 |
| 84 | 1605 | 2,2,4-Trimethyl-1,3-pentanediol diisobutyrate | 286 | C_16_H_30_O_4_ | 171,985 | 0.16 |
| 85 | 1609 | 2(3H)-Benzothiazolethione, 3-methyl- | 181 | C_8_H_7_NS_2_ | 394,667 | 0.36 |
| 82 | 1603 | Benzophenone | 182 | C_13_H_10_O | 48,025 | 0.04 |
| 96 | 1656 | 1-Tetradecanol | 214 | C_14_H_30_O | 805971 | 0.75 |
| 86 | 1769 | 2-Propenoic acid, tridecyl ester | 254 | C_16_H_30_O_2_ | 313,792 | 0.29 |
| 90 | 1746 | Heptadecane, 8-methyl- | 254 | C_18_H_38_ | 1,187,803 | 1.1 |
| 95 | 1731 | Ethanol, 2-(dodecyloxy)- | 230 | C1_4_H3_0_O_2_ | 1,958,476 | 1.81 |
| 90 | 1711 | Heptadecane | 240 | C_17_H_36_ | 1,045,152 | 0.97 |
| 90 | 1753 | Hexadecane, 2,6,10,14-tetramethyl- | 282 | C_20_H_42_ | 307,315 | 0.28 |
| 95 | 1755 | n-Pentadecanol | 228 | C_15_H_32_O | 198,544 | 0.18 |
| 86 | 1770 | Tridecanol, 2-ethyl-2-methyl- | 242 | C_16_H_34_O | 167,040 | 0.15 |
| 95 | 1612 | Hexadecane | 226 | C_16_H_34_ | 770,593 | 0.71 |
| 93 | 1908 | 1,2-Benzenedicarboxylic acid, bis(2-methylpropyl) ester | 278 | C_16_H_22_O_4_ | 464,182 | 0.43 |
| 93 | 1954 | n-Heptadecanol-1 | 256 | C_17_H_36_O | 52,185 | 0.05 |
| 87 | 1847 | 2-Heptadecanone | 254 | C_17_H_34_O | 94,454 | 0.09 |
| 86 | 1825 | 5,5-Diethylpentadecane | 268 | C_19_H_40_ | 172,925 | 0.16 |
| 85 | 1945 | 10-Methylnonadecane | 282 | C_20_H_42_ | 79,312 | 0.07 |
| 91 | 1968 | n-Hexadecanoic acid | 256 | C_16_H_32_O_2_ | 939,646 | 0.87 |
| 88 | 1810 | Octadecane | 254 | C_18_H_38_ | 1,537,685 | 1.42 |
| 85 | 1908 | 1,2-Benzenedicarboxylic acid, bis(2-methylpropyl) ester | 278 | C_16_H_22_O_4_ | 1,210,186 | 1.12 |
| 85 | 1852 | Heptadecane, 2,6,10,15-tetramethyl- | 296 | C_21_H_44_ | 451,716 | 0.42 |
| 96 | 1900 | 1-Nonadecene | 266 | C_19_H_38_ | 968,559 | 0.9 |
| 84 | 1890 | Methoxyacetic acid, 2-tetradecyl ester | 286 | C_17_H_34_O_3_ | 3,039,826 | 2.81 |
| 87 | 1945 | Nonadecane, 9-methyl- | 282 | C_20_H_42_ | 465615 | 0.43 |
| 88 | 2009 | Eicosane | 282 | C_20_H_42_ | 273021 | 0.25 |
| 90 | 2013 | Isopropyl palmitate | 298 | C1_9_H_38_O_2_ | 239206 | 0.22 |
| 86 | 2024 | 5,5-Diethylheptadecane | 296 | C_21_H_44_ | 132,125 | 0.12 |
| 85 | 2109 | Heneicosane | 296 | C_21_H_44_ | 169,660 | 0.16 |
| 94 | 2153 | n-Nonadecanol-1 | 284 | C_19_H_40_O | 110,004 | 0.1 |
| 80 | 2085 | 9-Octadecenoic acid (Z)-, methyl ester | 296 | C_19_H_36_O_2_ | 109,395 | 0.1 |
| 81 | 2011 | Octadecyl trifluoroacetate | 366 | C_20_H_37_F_3_O_2_ | 178,203 | 0.16 |
| 91 | 2077 | Methyl stearate | 298 | C1_9_H_38_O_2_ | 68,567 | 0.06 |
| 88 | 2045 | Pentadecane, 8-hexyl- | 296 | C2_1_H_44_ | 1,137,620 | 1.05 |
| 91 | 2167 | Octadecanoic acid | 284 | C_18_H_36_O_2_ | 424,451 | 0.39 |
| 89 | 2188 | 1-Dodecanol, 2-octyl- | 298 | C_20_H_42_O | 437,293 | 0.4 |
| 88 | 2026 | Hexadecane, 1-iodo- | 352 | C_16_H_33_I | 1,421,968 | 1.31 |
| 81 | 2124 | 5-Ethyl-5-methylnonadecane | 310 | C_22_H_46_ | 542,266 | 0.5 |
| 81 | 2102 | Hexadecanedinitrile | 248 | C_16_H_28_N_2_ | 497,862 | 0.46 |
| 83 | 2129 | Ethanol, 2-(hexadecyloxy)- | 286 | C_18_H_38_O_2_ | 812,322 | 0.75 |
| 81 | 2370 | Sulfurous acid, pentadecyl 2-propyl ester | 334 | C_18_H_38_O_3_S | 255,013 | 0.24 |
| 83 | 2370 | Oxalic acid, dodecyl 3,5-difluorophenyl ester | 370 | C_20_H_28_F_2_O_4_ | 178,075 | 0.16 |
| 85 | 2223 | 5-Butyl-5-ethylheptadecane | 324 | C_23_H_48_ | 80,383 | 0.07 |
| 95 | 2281 | Triethylene glycol monododecyl ether | 318 | C_18_H_38_O_4_ | 1,508,770 | 1.39 |
| 83 | 2442 | 2-methyltetracosane | 352 | C_25_H_52_ | 165,444 | 0.15 |
| 88 | 2451 | Behenic alcohol | 326 | C_22_H_46_O | 115,330 | 0.11 |
| 91 | 2407 | Tetracosane | 338 | C_24_H_50_ | 873,649 | 0.81 |
| 91 | 2556 | Tetraethylene glycol monododecyl ether | 362 | C_20_H_42_O_5_ | 575,872 | 0.53 |
| 90 | 2575 | Phthalic acid, di(6-methylhept-2-yl) ester | 390 | C_24_H_38_O_4_ | 1,307,181 | 1.21 |
| 84 | 2442 | 2-methyltetracosane | 352 | C_25_H_52_ | 415,813 | 0.38 |
| 84 | 2619 | Squalane | 422 | C_30_H_62_ | 100,233 | 0.09 |
| 84 | 2767 | Hexacosyl pentafluoropropionate | 528 | C_29_H_53_F_5_O_2_ | 131,919 | 0.12 |
| 92 | 2641 | 2-methylhexacosane | 380 | C_27_H_56_ | 610,411 | 0.56 |
| 85 | 2804 | Octacosane | 394 | C_28_H_58_ | 691,612 | 0.64 |
| 93 | 2914 | Squalene | 410 | C_30_H_50_ | 428,254 | 0.4 |
| 83 | 2840 | 2-methyloctacosane | 408 | C_29_H_60_ | 271,572 | 0.25 |
| 89 | 3103 | Hentriacontane | 436 | C_31_H_64_ | 1,291,784 | 1.19 |
| 83 | 3401 | Tetratriacontane | 478 | C_34_H_70_ | 74,059 | 0.07 |
| 92 | 3500 | Pentatriacontane | 492 | C_35_H_72_ | 742,253 | 0.69 |
| 87 | 3600 | Hexatriacontane | 506 | C_36_H_74_ | 227,989 | 0.21 |

Table S6. Compounds detected by GC-MS in the aerobic compartment of MBR-T (% match > 80%).

| **Similarity** | **Retention Index** | **Compound Name** | **Molecular Weight**  **(Da)** | **Chemical Formula** | | **Area** | | **Area**  **percentage**  **(%)** | |  |
| --- | --- | --- | --- | --- | --- | --- | --- | --- | --- | --- |
| 80 | 1070 | 2,6-Heptadien-1-ol, 2,4-dimethyl- (*) | 140 | | C_9_H_16_O | | 467,023 | | 0.46 | |
| 89 | 1185 | Undecane, 3,7-dimethyl- | 184 | | C_13_H_28_ | | 528,905 | | 0.52 | |
| 90 | 1150 | Undecane, 5-methyl- | 170 | | C_12_H_26_ | | 133,806 | | 0.13 | |
| 81 | 1086 | Decane, 5,6-dimethyl- | 170 | | C_12_H_26_ | | 291,676 | | 0.12 | |
| 81 | 1041 | 3-Ethyl-5-methyl-1-heptyn-3-ol | 154 | | C_10_H_18_O | | 128,135 | | 0.13 | |
| 83 | 1057 | 1-(2,4-Dimethyl-furan-3-yl)-ethanone (*) | 138 | | C_8_H_10_O_2_ | | 268,441 | | 0.26 | |
| 86 | 1178 | Cyclopropane, 1-ethyl-2-heptyl- | 168 | | C_12_H_24_ | | 155,183 | | 0.15 | |
| 92 | 1185 | Nonane, 5-(2-methylpropyl)- (*) | 184 | | C_13_H_28_ | | 319,626 | | 0.32 | |
| 90 | 1194 | 1-Heptanol, 2-propyl- (*) | 158 | | C_10_H_22_O | | 403,131 | | 0.40 | |
| 84 | 1057 | .alpha.-Hydroxyisobutyric acid, acetate | 146 | | C_6_H_10_O_4_ | | 287,584 | | 0.28 | |
| 84 | 1192 | Cyclooctanone, 2-methyl- | 140 | | C_9_H_16_O | | 752,159 | | 0.74 | |
| 93 | 1159 | 1-Nonanol | 144 | | C_9_H_20_O | | 313,690 | | 0.31 | |
| 82 | 1042 | 1,5-Heptadien-4-one, 3,3,6-trimethyl- | 152 | | C_10_H_16_O | | 92,451 | | 0.09 | |
| 84 | 1039 | 5-Ethyl-3-methylhept-1-en-4-ol (*) | 156 | | C_10_H_20_O | | 293,762 | | 0.29 | |
| 94 | 1285 | Dodecane, 4,6-dimethyl- (*) | 198 | | C_14_H_3_0 | | 478,195 | | 0.47 | |
| 90 | 1320 | Dodecane, 2,6,10-trimethyl- (*) | 212 | | C_15_H_32_ | | 146,164 | | 0.14 | |
| 92 | 1249 | Nonane, 5-butyl- | 184 | | C_13_H_28_ | | 450,605 | | 0.44 | |
| 89 | 1249 | Decane, 5-propyl- | 184 | | C_13_H_28_ | | 194,899 | | 0.19 | |
| 81 | 1277 | Undecanol-3 | 172 | | C_11_H_24_O | | 79,936 | | 0.08 | |
| 81 | 1331 | Propanoic acid, 2-methyl-, 3-hydroxy-2,4,4-trimethylpentyl ester | 216 | | C_12_H_24_O_3_ | | 31,442 | | 0.03 | |
| 95 | 1304 | 1-Tridecene (*) | 182 | | C_13_H_26_ | | 381,341 | | 0.38 | |
| 93 | 1313 | Tridecane (*) | 184 | | C_13_H_28_ | | 91,586 | | 0.09 | |
| 86 | 1438 | (p-Hydroxyphenyl)glyoxal (*) | 150 | | C_8_H_6_O_3_ | | 804,959 | | 0.79 | |
| 97 | 1457 | 1-Dodecanol (*) | 186 | | C_12_H_26_O | | 1,213,027 | | 1.2 | |
| 93 | 1555 | Phenol, 3,5-bis(1,1-dimethylethyl)- (*) | 206 | | C_14_H_22_O | | 3,565,292 | | 3.52 | |
| 84 | 1492 | 11-Methyldodecanol | 200 | | C_13_H_28_O | | 75,934 | | 0.07 | |
| 86 | 1446 | 2-Bromo dodecane (*) | 248 | | C_12_H_25_Br | | 328,875 | | 0.32 | |
| 86 | 1413 | Tetradecane (*) | 198 | | C_14_H_30_ | | 124,371 | | 0.12 | |
| 95 | 1512 | Pentadecane (*) | 212 | | C_15_H_32_ | | 495,074 | | 0.65 | |
| 95 | 1656 | 1-Tetradecanol (*) | 214 | | C_14_H_30_O | | 1,188,778 | | 1.17 | |
| 91 | 1746 | Heptadecane, 8-methyl- (*) | 254 | | C_18_H_38_ | | 908,666 | | 0.9 | |
| 95 | 1731 | Ethanol, 2-(dodecyloxy)- (*) | 230 | | C_14_H_30_O_2_ | | 2,592,728 | | 2.56 | |
| 90 | 1753 | Hexadecane, 2,6,10,14-tetramethyl- | 282 | | C_20_H_42_ | | 318,712 | | 0.31 | |
| 90 | 1612 | Hexadecane (*) | 226 | | C_16_H_34_ | | 903,201 | | 0.89 | |
| 81 | 1688 | Octane, 1,1'-oxybis- | 242 | | C_16_H_34_O | | 101,432 | | 0.10 | |
| 89 | 1645 | 2-Bromotetradecane (*) | 276 | | C_14_H_29_Br | | 130,934 | | 0.13 | |
| 84 | 1627 | 5,5-Diethyltridecane | 240 | | C_17_H_36_ | | 111,176 | | 0.11 | |
| 85 | 1770 | Tridecanol, 2-ethyl-2-methyl- (*) | 242 | | C_16_H_34_O | | 258,864 | | 0.26 | |
| 86 | 1753 | Hexadecane, 2,6,11,15-tetramethyl- (*) | 282 | | C_20_H_42_ | | 188,053 | | 0.19 | |
| 95 | 1755 | n-Pentadecanol (*) | 228 | | C_15_H_32_O | | 1,104,603 | | 1.09 | |
| 93 | 1746 | Heptadecane, 8-methyl- | 254 | | C_18_H_38_ | | 814,628 | | 0.8 | |
| 85 | 1805 | p-Toluic acid, 2-ethylhexyl ester | 224 | | C_16_H_24_ O_2_ | | 107,108 | | 0.11 | |
| 94 | 1908 | 1,2-Benzenedicarboxylic acid, bis(2-methylpropyl) ester (*) | 278 | | C_16_H_22_O_4_ | | 717,167 | | 1.04 | |
| 93 | 1954 | n-Heptadecanol-1 (*) | 256 | | C_17_H_36_O | | 139,978 | | 0.14 | |
| 92 | 1852 | Heptadecane, 2,6,10,15-tetramethyl- (*) | 296 | | C_21_H_44_ | | 235,410 | | 0.23 | |
| 86 | 1847 | 2-Heptadecanone (*) | 254 | | C_17_H_34_O | | 135,263 | | 0.13 | |
| 80 | 1930 | Ethanol, 2-(tetradecyloxy)- | 258 | | C_16_H_34_O_2_ | | 3,019,637 | | 2.98 | |
| 81 | 1910 | Nonadecane (*) | 268 | | C_19_H_40_ | | 195,166 | | 0.19 | |
| 93 | 1968 | n-Hexadecanoic acid (*) | 256 | | C_16_H_32_O_2_ | | 1,136,653 | | 1.12 | |
| 88 | 1825 | 5,5-Diethylpentadecane (*) | 268 | | C_19_H_40_ | | 1,361,658 | | 1.34 | |
| 96 | 1900 | 1-Nonadecene (*) | 266 | | C_19_H_38_ | | 1,088,310 | | 1.07 | |
| 84 | 1890 | Methoxyacetic acid, 2-tetradecyl ester (*) | 286 | | C_17_H_34_O_3_ | | 3,658,108 | | 3.61 | |
| 88 | 2045 | Pentadecane, 8-hexyl- (*) | 296 | | C_21_H_44_ | | 233,851 | | 0.23 | |
| 85 | 2013 | Isopropyl palmitate (*) | 298 | | C_19_H_38_O_2_ | | 239,393 | | 0.24 | |
| 85 | 2024 | 5,5-Diethylheptadecane (*) | 296 | | C_21_H_44_ | | 131,666 | | 0.13 | |
| 92 | 2053 | 1-Octadecanol | 270 | | C_18_H_38_O | | 159,890 | | 0.16 | |
| 81 | 2085 | 9-Octadecenoic acid, methyl ester, (E)- (*) | 296 | | C_19_H_36_O_2_ | | 137,366 | | 0.14 | |
| 82 | 2178 | 2(3H)-Furanone, dihydro-5-tetradecyl- | 282 | | C_18_H_34_O_2_ | | 43,518 | | 0.04 | |
| 90 | 2077 | Methyl stearate (*) | 298 | | C_19_H_38_O_2_ | | 100,326 | | 0.1 | |
| 90 | 2009 | Eicosane | 282 | | C2_0_H_42_ | | 975,811 | | 0.96 | |
| 89 | 2167 | Octadecanoic acid (*) | 284 | | C_18_H_36_O_2_ | | 464,273 | | 0.46 | |
| 88 | 2188 | 1-Dodecanol, 2-octyl- (*) | 298 | | C_20_H_42_O | | 443,877 | | 0.44 | |
| 88 | 2109 | Heneicosane (*) | 296 | | C_21_H_44_ | | 1,201,625 | | 1.19 | |
| 90 | 2153 | n-Nonadecanol-1 (*) | 284 | | C_19_H_40_O | | 533,675 | | 0.53 | |
| 91 | 2045 | Pentadecane, 8-hexyl- (*) | 296 | | C_21_H_44_ | | 1,159,295 | | 1.14 | |
| 91 | 2006 | Diethylene glycol monododecyl ether | 274 | | C_16_H_34_O_3_ | | 1,026,150 | | 1.01 | |
| 86 | 2370 | Sulfurous acid, pentadecyl 2-propyl ester (*) | 334 | | C_18_H_38_O_3_S | | 419,106 | | 0.41 | |
| 85 | 2223 | 5-Butyl-5-ethylheptadecane (*) | 324 | | C_23_H_48_ | | 291,356 | | 0.29 | |
| 94 | 2281 | Triethylene glycol monododecyl ether (*) | 318 | | C_18_H_38_O_4_ | | 1,928,738 | | 1.90 | |
| 82 | 2252 | 1-Eicosanol | 298 | | C_20_H_42_O | | 81,858 | | 0.08 | |
| 91 | 2556 | Tetraethylene glycol monododecyl ether (*) | 362 | | C_20_H_42_O_5_ | | 513,130 | | 0.74 | |
| 90 | 2575 | Phthalic acid, di(6-methylhept-2-yl) ester | 390 | | C_24_H_38_O_4_ | | 902,074 | | 0.89 | |
| 84 | 2442 | 2-methyltetracosane (*) | 352 | | C_25_H_52_ | | 327,625 | | 0.32 | |
| 91 | 2407 | Tetracosane (*) | 338 | | C_24_H_50_ | | 1,155,702 | | 1.14 | |
| 85 | 2668 | Sulfurous acid, octadecyl 2-propyl ester | 376 | | C_21_H_44_O_3_S | | 74,663 | | 0.07 | |
| 82 | 2650 | n-Tetracosanol-1 | 354 | | C_24_H_50_O | | 117,227 | | 0.12 | |
| 92 | 2641 | 2-methylhexacosane (*) | 380 | | C_27_H_56_ | | 417,577 | | 0.42 | |
| 84 | 2619 | Squalane (*) | 422 | | C_30_H_62_ | | 170,290 | | 0.17 | |
| 82 | 2914 | Squalene (*) | 410 | | C_30_H_50_ | | 105,094 | | 0.10 | |
| 90 | 2840 | 2-methyloctacosane (*) | 408 | | C_29_H_60_ | | 469,298 | | 0.46 | |
| 91 | 3047 | Octacosanol | 410 | | C_28_H_58_O | | 397,291 | | 0.39 | |

Note:

1. (*) represents the compound that was also detected in the anoxic compartment of MBR-T.

Table S7. Compounds detected by GC-MS in the MBR-T permeate (% match > 80%).

| **Similarity** | | **Retention Index** | | **Compound Name** | | **Molecular Weight (Da)** | **Chemical Formula** | | **Area** | | **Percentage**  **(%)** | **Removal (%)** |  |
| --- | --- | --- | --- | --- | --- | --- | --- | --- | --- | --- | --- | --- | --- |
| 89 | 1105 | | 1-Undecene | | 154 | | C_11_H_22_ | 150,224 | | 0.18 | | N.A. |  |
| 92 | 1185 | | Nonane, 3-methyl-5-propyl- | | 184 | | C_13_H_28_ | 458,593 | | 0.54 | | N.A. |  |
| 89 | 1194 | | 1-Heptanol, 2-propyl- (**) | | 158 | | C_10_H_22_O | 525,331 | | 0.62 | | <0 |  |
| 87 | 1018 | | Nonane, 2-methyl-3-methylene- | | 154 | | C_11_H_22_ | 412,240 | | 0.48 | | N.A. |  |
| 83 | 1057 | | 1-(2,4-Dimethyl-furan-3-yl)-ethanone | | 138 | | C_8_H_10_O_2_ | 355,637 | | 0.42 | | <0 |  |
| 81 | 1091 | | 3-Methyl-2-butenoic acid, cyclobutyl ester | | 154 | | C_9_H_14_O_2_ | 139,270 | | 0.16 | | N.A. |  |
| 94 | 1285 | | Dodecane, 4,6-dimethyl- (**) | | 198 | | C_14_H_30_ | 632,072 | | 0.74 | | <0 |  |
| 91 | 1320 | | Dodecane, 2,6,10-trimethyl- (**) | | 212 | | C_15_H_32_ | 176,045 | | 0.21 | | <0 |  |
| 86 | 1285 | | 2,3-Dimethyldodecane | | 198 | | C_14_H_30_ | 192,200 | | 0.23 | | N.A. |  |
| 92 | 1249 | | Nonane, 5-butyl- (*) | | 184 | | C1_3_H_28_ | 577,836 | | 0.68 | | <0 |  |
| 91 | 1304 | | 1-Tridecene (**) | | 182 | | C_13_H_26_ | 168,126 | | 0.2 | | 55.9 |  |
| 85 | 1438 | | (p-Hydroxyphenyl)glyoxal (**) | | 150 | | C_8_H_6_O_3_ | 359,202 | | 0.42 | | 55.4 |  |
| 96 | 1457 | | 1-Dodecanol (**) | | 186 | | C_12_H_26_O | 741,166 | | 0.87 | | 38.9 |  |
| 80 | 1469 | | 2-Pentanone, 4-(1,3,3-trimethyl-7-oxabicyclo[4.1.0]hept-2-yl)- | | 224 | | C_14_H_24_O_2_ | 429,041 | | 0.5 | | N.A. |  |
| 93 | 1555 | | Phenol, 3,5-bis(1,1-dimethylethyl)- (**) | | 206 | | C_14_H_22_O | 3,706,053 | | 4.36 | | <0 |  |
| 89 | 1413 | | Tetradecane (**) | | 198 | | C_14_H_30_ | 157,514 | | 0.19 | | <0 |  |
| 85 | 1448 | | Tetradecane, 4-methyl- | | 212 | | C_15_H_32_ | 193,765 | | 0.23 | | N.A. |  |
| 94 | 1502 | | 1-Pentadecene | | 210 | | C_15_H_30_ | 268,754 | | 0.32 | | N.A. |  |
| 89 | 1605 | | Propanoic acid, 2-methyl-, 1-(1,1-dimethylethyl)-2-methyl-1,3-propanediyl ester | | 286 | | C_16_H_30_O_4_ | 242,704 | | 0.29 | | N.A. |  |
| 97 | 1656 | | 1-Tetradecanol (**) | | 214 | | C_14_H_30_O | 556,484 | | 0.65 | | 53.2 |  |
| 84 | 1670 | | Dodecyl acrylate | | 240 | | C_15_H_28_O_2_ | 186,902 | | 0.22 | | N.A. |  |
| 95 | 1731 | | Ethanol, 2-(dodecyloxy)- (**) | | 230 | | C_14_H_30_O_2_ | 1,521,970 | | 1.79 | | 41.3 |  |
| 91 | 1746 | | Heptadecane, 8-methyl- (**) | | 254 | | C_18_H_38_ | 1,102,655 | | 1.3 | | <0 |  |
| 85 | 1770 | | Tridecanol, 2-ethyl-2-methyl- (**) | | 242 | | C_16_H_34_O | 140,104 | | 0.16 | | 45.9 |  |
| 85 | 1753 | | Hexadecane, 2,6,10,14-tetramethyl- | | 282 | | C_20_H_42_ | 218,687 | | 0.26 | | 31.4 |  |
| 93 | 1755 | | n-Pentadecanol (**) | | 228 | | C_15_H_32_O | 486,213 | | 0.57 | | 56.0 |  |
| 92 | 1908 | | 1,2-Benzenedicarboxylic acid, bis(2-methylpropyl) ester (**) | | 278 | | C_16_H_22_O_4_ | 166,860 | | 0.2 | | 76.7 |  |
| 87 | 1847 | | 2-Heptadecanone (**) | | 254 | | C_17_H_34_O | 65,862 | | 0.08 | | 51.3 |  |
| 86 | 1945 | | 10-Methylnonadecane | | 282 | | C_20_H_42_ | 1,888,531 | | 2.22 | | N.A. |  |
| 88 | 1852 | | Heptadecane, 2,6,10,15-tetramethyl- (**) | | 296 | | C_21_H_44_ | 201,975 | | 0.24 | | 14.2 |  |
| 85 | 1846 | | Octadecane, 5-methyl- | | 268 | | C_19_H_40_ | 98,214 | | 0.12 | | N.A. |  |
| 92 | 1968 | | n-Hexadecanoic acid (**) | | 256 | | C_16_H_32_O_2_ | 605,899 | | 0.71 | | 46.7 |  |
| 94 | 2071 | | Nonadecyl pentafluoropropionate | | 430 | | C_22_H_39_F_5_O_2_ | 582,991 | | 0.69 | | N.A. |  |
| 93 | 2006 | | Diethylene glycol monododecyl ether (*) | | 274 | | C_16_H_34_O_3_ | 2,572,374 | | 3.02 | | <0 |  |
| 89 | 2109 | | Heneicosane (**) | | 296 | | C_21_H_44_ | 366,707 | | 0.43 | | 69.5 |  |
| 89 | 2045 | | Pentadecane, 8-hexyl- (**) | | 296 | | C_21_H_44_ | 235,864 | | 0.28 | | <0 |  |
| 86 | 2013 | | Isopropyl palmitate (**) | | 298 | | C_19_H_38_O_2_ | 211,078 | | 0.25 | | 11.8 |  |
| 92 | 2167 | | Octadecanoic acid (**) | | 284 | | C_18_H_36_O_2_ | 235,671 | | 0.28 | | 49.2 |  |
| 88 | 2188 | | 1-Dodecanol, 2-octyl- (**) | | 298 | | C_20_H_42_O | 425,509 | | 0.5 | | 4.1 |  |
| 82 | 2102 | | Hexadecanedinitrile | | 248 | | C_16_H_28_N_2_ | 319,796 | | 0.38 | | N.A. |  |
| 85 | 2328 | | Ethanol, 2-(octadecyloxy)- | | 314 | | C_20_H_42_O_2_ | 715,393 | | 0.84 | | N.A. |  |
| 86 | 2230 | | Heneicosyl heptafluorobutyrate | | 508 | | C_25_H_43_F_7_O_2_ | 248,943 | | 0.29 | | N.A. |  |
| 83 | 2178 | | 2(3H)-Furanone, dihydro-5-tetradecyl- (*) | | 282 | | C_18_H_34_O_2_ | 70,088 | | 0.08 | | <0 |  |
| 83 | 2228 | | 9-Octadecenamide, (Z)- | | 281 | | C_18_H_35_NO | 373,734 | | 0.44 | | N.A. |  |
| 91 | 2407 | | Tetracosane (**) | | 338 | | C_24_H_50_ | 1,680,590 | | 1.98 | | <0 |  |
| 88 | 2505 | | Phthalic acid, 2-ethylhexyl isohexyl ester | | 362 | | C_22_H_34_O_4_ | 497,504 | | 0.58 | | N.A. |  |
| 86 | 2641 | | 2-methylhexacosane (**) | | 380 | | C_27_H_56_ | 642,978 | | 0.76 | | <0 |  |
| 85 | 2619 | | Squalane (**) | | 422 | | C_30_H_62_ | 103,439 | | 0.12 | | 39.3 |  |
| 92 | 2840 | | 2-methyloctacosane (**) | | 408 | | C_29_H_60_ | 1,004,544 | | 1.18 | | <0 |  |
| 92 | 2914 | | Squalene (**) | | 410 | | C_30_H_50_ | 269,948 | | 0.32 | | <0 |  |

Note:

1. (*) represents the compound that was also detected in the aerobic compartment of MBR-T.
2. (**) represents the compound that was also detected in the anoxic and aerobic compartments of MBR-T.
